# Radical Scavenging Could Answer the Challenge Posed by Electron–Electron Dipolar Interactions in the Cryptochrome Compass Model

**DOI:** 10.1021/jacsau.1c00332

**Published:** 2021-10-05

**Authors:** Nathan Sean Babcock, Daniel R. Kattnig

**Affiliations:** † Quantum Biology Laboratory, Howard University, 2400 Sixth Street NW, Washington District of Columbia, 20059, United States of America; ‡ Living Systems Institute and Department of Physics University of Exeter, Stocker Road, Exeter, EX4 4QD, United Kingdom

**Keywords:** magnetoreception, radical pair mechanism, electron−electron dipolar coupling, three-radical effects, cryptochrome

## Abstract

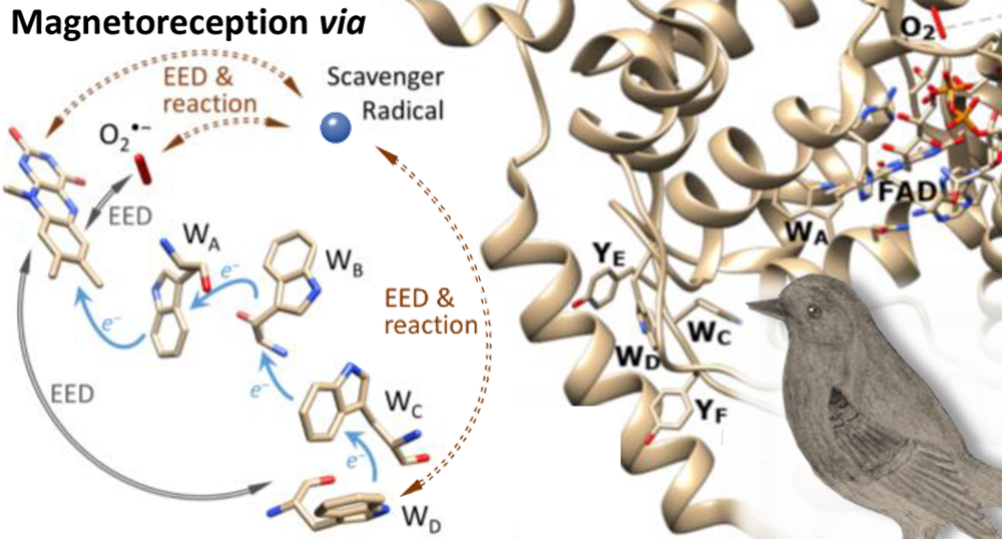

Many birds are endowed with a visual magnetic sense that may exploit magnetosensitive radical recombination processes in the protein cryptochrome. In this widely accepted but unproven model, geomagnetic sensitivity is suggested to arise from variations in the recombination rate of a pair of radicals, whose unpaired electron spins undergo coherent singlet–triplet interconversion in the geomagnetic field by coupling to nuclear spins via hyperfine interactions. However, simulations of this conventional radical pair mechanism (RPM) predicted only tiny magnetosensitivities for realistic conditions because the RPM’s directional sensitivity is strongly suppressed by the intrinsic electron–electron dipolar (EED) interactions, casting doubt on its viability as a magnetic sensor. We show how this RPM-suppression problem is overcome in a three-radical system in which a third “scavenger” radical reacts with one member of the primary pair. We use this finding to predict substantial magnetic field effects that exceed those of the RPM in the presence of EED interactions in animal cryptochromes.

## Introduction

Various animals exhibit a light-dependent axial magnetic sense that is responsive to the inclination (but not the polarity) of the Earth’s magnetic field. These organisms include different types of birds, as well as insects and amphibians.^1−4^ Although magnetic sensing is widespread throughout the animal kingdom (where various magnetoreceptive mechanisms are employed), axial magnetoreception has attracted attention because it is hypothesized to rely on coherent quantum dynamics which control a chemical step.^2,5−7^ The actual sensor has, however, so far eluded discovery, and so, the reaction mechanism remains unclear and subject to controversy.^4^ This issue is complicated by the challenge of corroborating physicochemical models with relevant behavioral observations in vivo.^8^ This has led to a proliferation of models^9−16^ inspired by phenomenological observations.^17−23^

In the most widely accepted model of the inclination compass, sensing is actuated by the radical–pair mechanism (RPM).^2,15,16^ This long-standing model mechanism is known to govern various magnetic-field-sensitive kinetic effects in spin chemistry.^24^ Conventionally, the model assumes two *geminate* molecular radicals, “born together” from closed-shell reactants, possibly by photoexcitation or a light-independent reaction cascade.^2^ This radical pair initially comprises two physically separated, spin-correlated molecules in a shared “singlet” (i.e., zero) spin angular momentum state if born from the diamagnetic precursor (without intersystem crossing). Starting out from this pure singlet state, the radical pair can evolve in time under local magnetic interactions, which modulate its singlet/triplet character, and thus its likelihood of recombination. These dynamics are mediated by the Zeeman effect, in conjunction with the local magnetic field variations resulting from the nuclear hyperfine interactions. Thus, hyperfine interactions with local magnetic nuclei are the main driver of singlet–triplet interconversion in the RPM. The anisotropy of the hyperfine interactions (i.e., the electron–nuclear dipole coupling) imprints directionality on the magnetosensitivity of this process, providing the theoretical basis for the RPM-based inclination compass.

**Figure 1:**
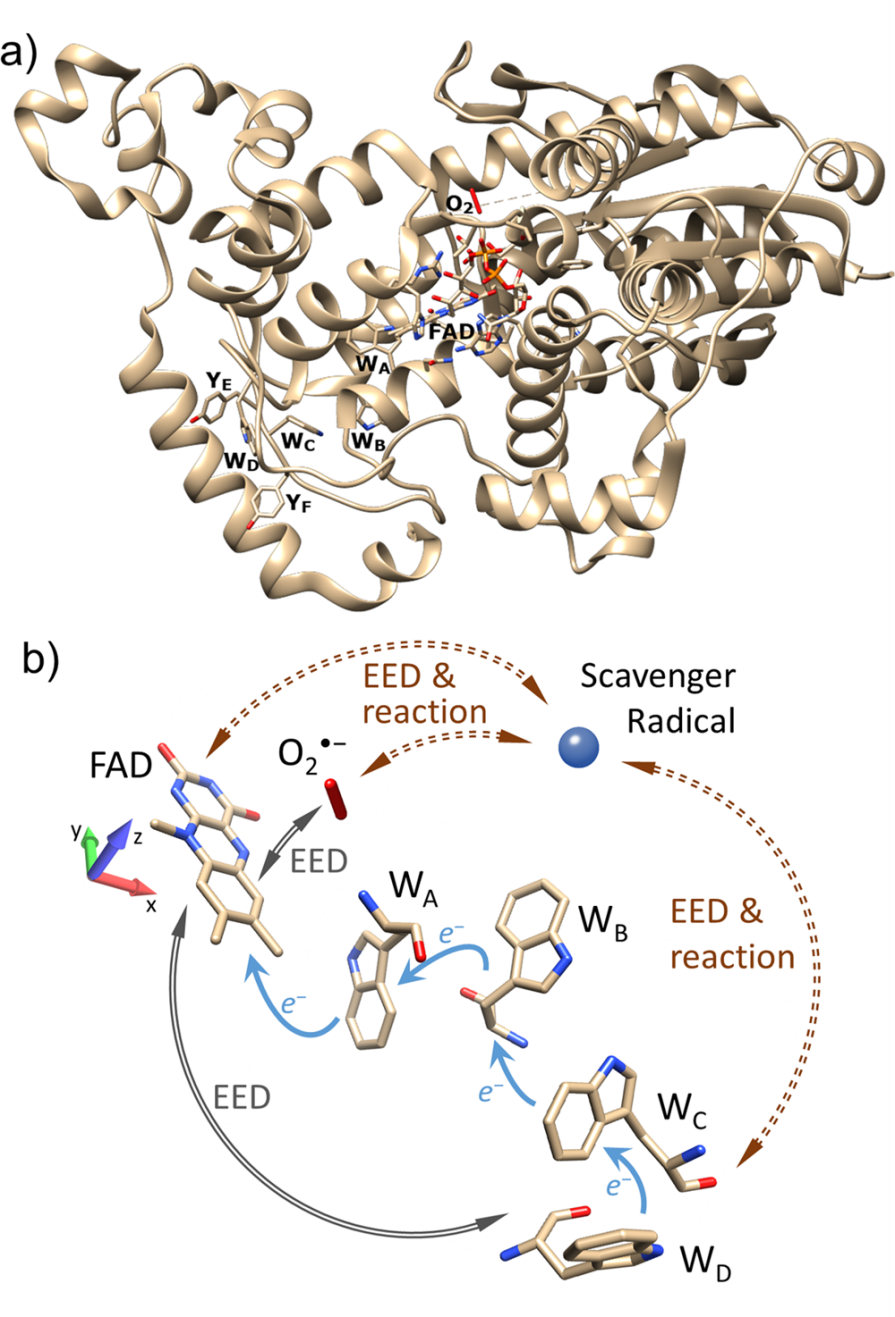
(a) Structure of *Columba livia* (pigeon) cryptochrome 4, *Cl*Cry4 (PDB: 6PU0).^57^ Labels indicate the FAD prosthetic group, the Trp tetrad, potential Tyr successors, and molecular oxygen in the position of the hypothetical Z_far_ radical used in our simulations. The loop covering the FAD that was not resolved in the crystal structure has been omitted. (b) Enlarged representation of the pertinent ET pathway constituted by the tryptophan tetrad and relevant radical sites. Both photoreduction and reoxidation pathways are shown simultaneously although they correspond to different stages of the reaction cycle.

According to the conventional RPM model of magnetoreception, chemical sensitivities to the amplitude and direction of an ambient magnetic field arise in the photoreceptor protein cryptochrome (Cry, Figure 1).^15^ In this model, the magnetic-field dependent, coherent spin dynamics modulate the proportion of radical pairs that recombine with respect to those that “escape” recombination, where those that escape are assumed to generate a structurally distinct signaling state (perhaps by adopting a different molecular conformation).^25^ In this way, the RPM forms the basis for a sensor that is insensitive to field polarity, responsive to a narrow (but adjustable) range of magnetic field intensities, light-dependent (though the magnetosensitive step may be light-independent,^20^ cf., below), and disrupted by weak radio frequency (rf) electromagnetic fields.^2,15,26^ These properties are thought to distinguish it from the magnetic particle-mediated sense.^27^ Specifically, the prediction of rf-interference has been borne out in many experiments, for example, on birds,^19,28−30^ crustaceans,^31^ fruit flies,^32^ and plants.^33^

Cryptochrome was suggested as a candidate magnetic receptor by Ritz et al.,^15^ because it had been found in mammalian eyes and was known to undergo the right kind of light-dependent radical reaction (as a protein in the cryptochrome/photolyase family^34^). Cryptochromes have since been found in birds’ and other species’ eyes.^35,36^ Interestingly, for some cryptochromes, there is strong evidence to implicate their role in the regulation of avian seasonal migration,^37,38^ a function beyond their activity as the circadian governor.^36^ Furthermore, evidence has accumulated to show that cryptochrome plays a critical role in magnetosensation in fruit flies, where gene knockout experiments have demonstrated that cryptochrome-deficient flies lose their magnetic sense.^22^ Yet, it remains unclear whether cryptochrome is the primary magnetoreceptor, or a downstream signal transducer.

**Scheme 1:**
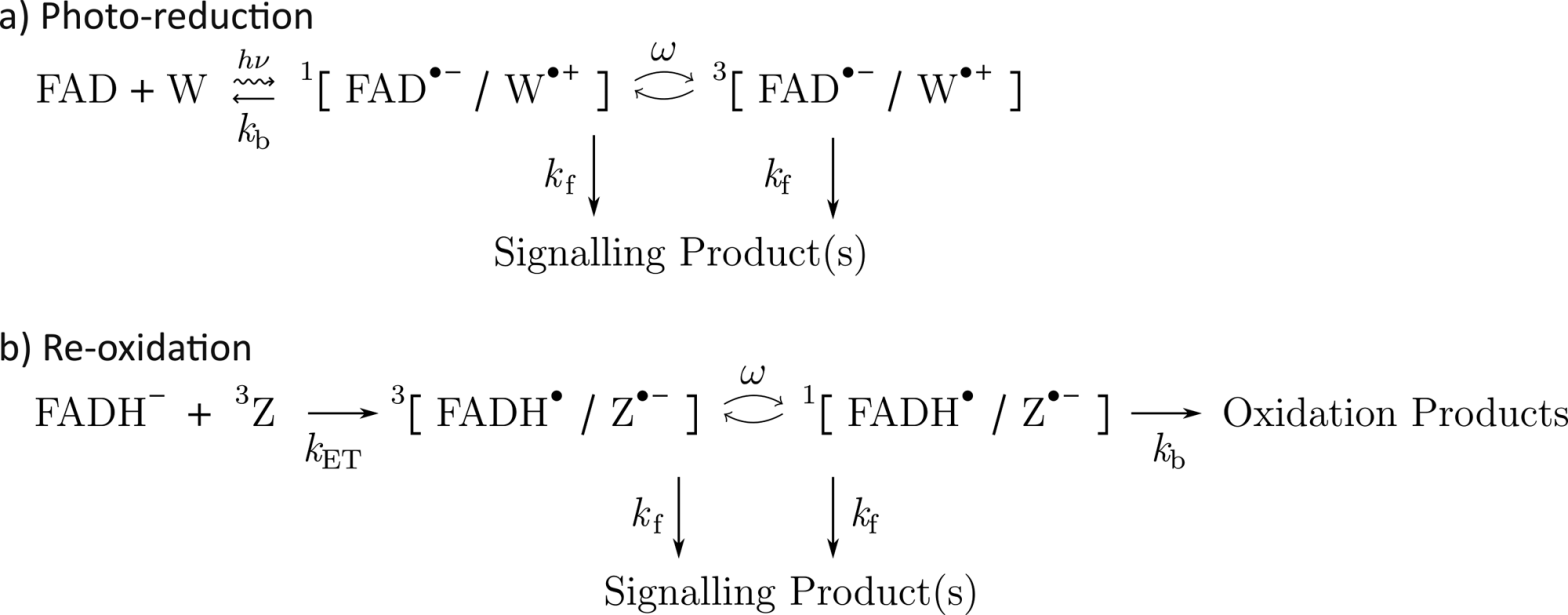
(a) Conventional RPM Model of Magnetoreception Employing [FAD^•–^/W^•+^] Produced via Photoexcitation (Typically Involving Blue Light) and (b) Alternative Radical-Pair-Based Scheme of Magnetoreception Employing [FADH^•^/Z^•–^] Produced via ET from FADH^–^ to Z (Rate *k*_ET_) The radical pair is born as a singlet and undergoes coherent singlet-triplet interconversion (labeled ω) before recombining (as a singlet at rate *k*_b_) or initiating signalling (independent of the total spin at a rate *k*_f_). The radical pair is assumed to be born in the triplet state (in line with Z’s putative identity as molecular oxygen), and undergoes coherent singlet-triplet interconversion before it reacts either to form a closed-shell oxidation product (e.g., a hydroperoxide of FAD) or to initiate the resulting signalling cascade.

In vitro, the cryptochrome-cofactor flavin adenine dinucleotide (FAD), is photoreduced in a reaction involving spin-conserving sequential electron transfer (ET) processes along a structurally conserved triad (in plants) or tetrad (in animals) of tryptophan residues (respectively, labeled W_A_, W_B_, W_C_, W_D_)^39−44^ as depicted in Figure 1. Thus, cryptochrome-compass models are often based on a flavin/tryptophan radical pair [FAD^•–^/W^•+^], born out of the photoreduction of the fully oxidized (quinone) FAD by the surface-exposed tryptophan W_C_ via the Trp chain (Scheme 1a).^2^ Indeed, there is growing in vitro evidence that confirms the presence of mT-scale magnetic field effects (MFEs) related to this photoreduction pathway in isolated cryptochromes^41−43^ and photolyases,^45,46^ and even μT-scale MFEs have been reported in cryptochrome.^47^ The isolated cryptochrome 4 of the Europen Robin has recently been found to be more magnetosensitive to mT-magnetic fields in vitro than comparable cryptochromes of nonmigratory bird species.^44^ On the other hand, some studies have called the [FAD^•–^/W^•+^] scheme into question as a compass mechanism in vivo,^17−20,22^ hinting at an alternative explanation of axial magnetosensitivity in vivo, in terms of an ultimately light-driven process that incorporates a light-independent magnetosensitive step.

An alternative reaction scheme involves reoxidation of the fully photoreduced (hydroquinone) FADH^–^ by molecular oxygen.^14,48^ This gives rise to a semiquinone flavin/superoxide radical pair [FADH^•^/O_2_^•–^] (Scheme 1b).^14,49^ Based on its distribution of hyperfine interactions alone, the [FADH^•^/O_2_^•–^] pair has been implicated with the potential for large directional sensitivity, resulting from the complete lack of magnetic nuclei in O_2_^•–^ (referred to as the reference–probe model).^10,50^ However, although O_2_^•–^ may be optimal in this respect, an unbound superoxide radical would suffer rapid decoherence while tumbling in solution, and therefore might not be practical.^13,51^ To avoid this issue, it became customary to assume a hypothetical radical denoted Z^•–^ that is devoid of hyperfine interactions,^10^ yet bestowed with the quality of slow spin relaxation.

In both these RPM models of magnetoreception (Scheme 1), the directional magnetosensitivity arises from the matched interplay of predominantly hyperfine and Zeeman interactions, and the difference in the chemical reactivities of the singlet and triplet states. Theory predicts modest (e.g., Scheme 1a under realistic conditions)^52,53^ to considerable (e.g., Scheme 1b without spin relaxation)^10,50^ directional MFEs. However, problems arise when inter-radical couplings, that is, exchange and EED coupling, modify the spin dynamics. These interactions limit the low-field magnetosensitivity by lifting the approximate zero-field degeneracy of the singlet and triplet states, thereby inhibiting the hyperfine-driven singlet–triplet interconversion on which the MFEs rely.^54,55^ As a consequence, the conventional RPM model loses magnetosensitivity if its radical pair is formed too close together. Yet, its magnetosensitivity is also lost if the pair is formed too far apart, for lack of appreciable recombination (as *k*_b_ → 0, precluding the discrimination of spin states). Note further that while the EED-coupling is anisotropic, it alone does not induce a directional MFE in a radical pair.^55,56^

EED interactions are unavoidably significant for radical pairs bound to cryptochrome,^55^ although electronic exchange was found to be negligible in studies of related compounds.^58^ For example, if we assume that the magnetic sensitivity arises principally from a radical pair containing the third tryptophan (W_C_) of the Trp chain (for which the radical pair separation is about 1.8 nm),^43^ then the magnitude of the EED coupling is |*D*| = 14 MHz. This coupling is at least comparable to the hyperfine couplings in FAD^•–^ and W^•+^ and exceeds the Zeeman interaction with the geomagnetic field (about 50 μT or 1.4 MHz). Thus, we can anticipate a non-negligible influence of EED coupling on the spin dynamics, which, as laid out above, is known to diminish the nuclear hyperfine-mediated RPM.^54,55^

This problem has been largely ignored in theoretical works that focused on the preferable, if unrealistic, scenario of negligible inter-radical interactions.^52,59−61^ One study to address the EED problem predicted that deleterious effects of the dipolar coupling *D* and exchange coupling *J* might be mitigated by meeting certain equal-but-opposite energy-matching conditions, which would cause *D* and *J* to partially eliminate each other. This mutual “*J*/*D* cancellation” effect would allow the essential zero-field degeneracies to be partially restored.^62^ However, a subsequent analysis predicted that *J*/*D* cancellation is unlikely to enable RPM-mediated MFEs in cryptochrome or other flavin-based radical-pair systems.^55^ In that study, the RPM’s magnetic sensitivity was shown to be suppressed by inter-radical interactions at the matching conditions and, for that matter, any other plausible values of the *J* coupling. This raises questions about whether it is feasible for the RPM to rationalize crytochrome-mediated magnetoreception, and invites the possibility that the RPM models shown in Scheme 1 may not be sufficient to rationalize such an exquisitely sensitive compass.

**Scheme 2:**
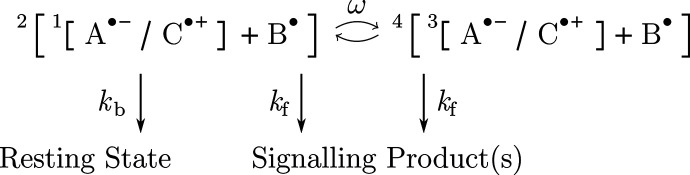
Bystander-Enabled Interconversion Mechanism, Wherein the Initial Anion–Cation [A^•–^/C^•+^] Radical Pair Is Produced in a Singlet or Triplet State via Photo-excitation or Some Other Reaction (Not Shown) In either case, the radical pair undergoes singlet–triplet interconversion, which is catalyzed by the presence of a bystander B^•^ before the primary radical pair either recombines (*k*_b_) or gives way to the signalling state (*k*_f_).^55^.

Speaking broadly, hyperfine coupling is not necessary to mediate the magnetic sensitivity of a radical recombination in a weak field.^56^ Rather, the necessary field-dependent singlet–triplet interconversion may be enabled by a radical pair coupling to a third radical via the seemingly intrusive EED interaction itself, as shown in Scheme 2.^56^ To this effect, we recently performed a theoretical analysis examining the influence of a third radical on the cryptochrome magnetoreceptor in the presence of all relevant EED interactions (in addition to hyperfine interactions).^55^ That generalized “Radical Trio Mechanism” (R3M) did indeed predict modest-but-significant MFEs in a cryptochrome magnetoreceptor in the presence of a third, catalytic “bystander” radical B^•^. However, although the presence of a bystander was found to boost the MFE anisotropy of a [FAD^•–^/W_D_^•+^] sensor by as much as factor of 20, via the EED coupling, the MFE did not exceed 3% for recombination parameters obtained on experimental evidence where the bystander radical was assumed unreactive.^55^

The introduction of a third nearby radical presents another previously unexplored possibility that remains as a means to tackle the EED problem: One member of the primary pair could react with the third radical to form a stable closed-shell product, rather than recombining with its twin to recover the ground-state protein configuration (or a new singlet-state reaction product). Indeed, past studies predicted a strong enhancement of the anisotropic MFEs by introducing the “scavenging” of one member of the geminate pair as it reacted with a “scavenger” radical S^•^.^63,64^ Crucially, this scavenging mechanism lifts the requirement that the primary-pair radicals be close enough together to enable sufficiently fast recombination. The problem has been debated in the context of Scheme 1, where the radical pair is assumed to include the terminal tryptophan of the tetrad (W_D_), which is too distant from the flavin to recombine on the required time scale.^63^ On the other hand, a scavenger was predicted to effectively “immunize” spin dynamics against the destructive effects of fast relaxation in one of the radicals, lending credibility to models implicating O_2_^•–^.^64^ However, both of those studies investigated only toy-model versions of the RPM in the presence of a scavenger radical, *without* including inter-radical interactions such as the invasive EED coupling.^63,64^

**Scheme 3:**
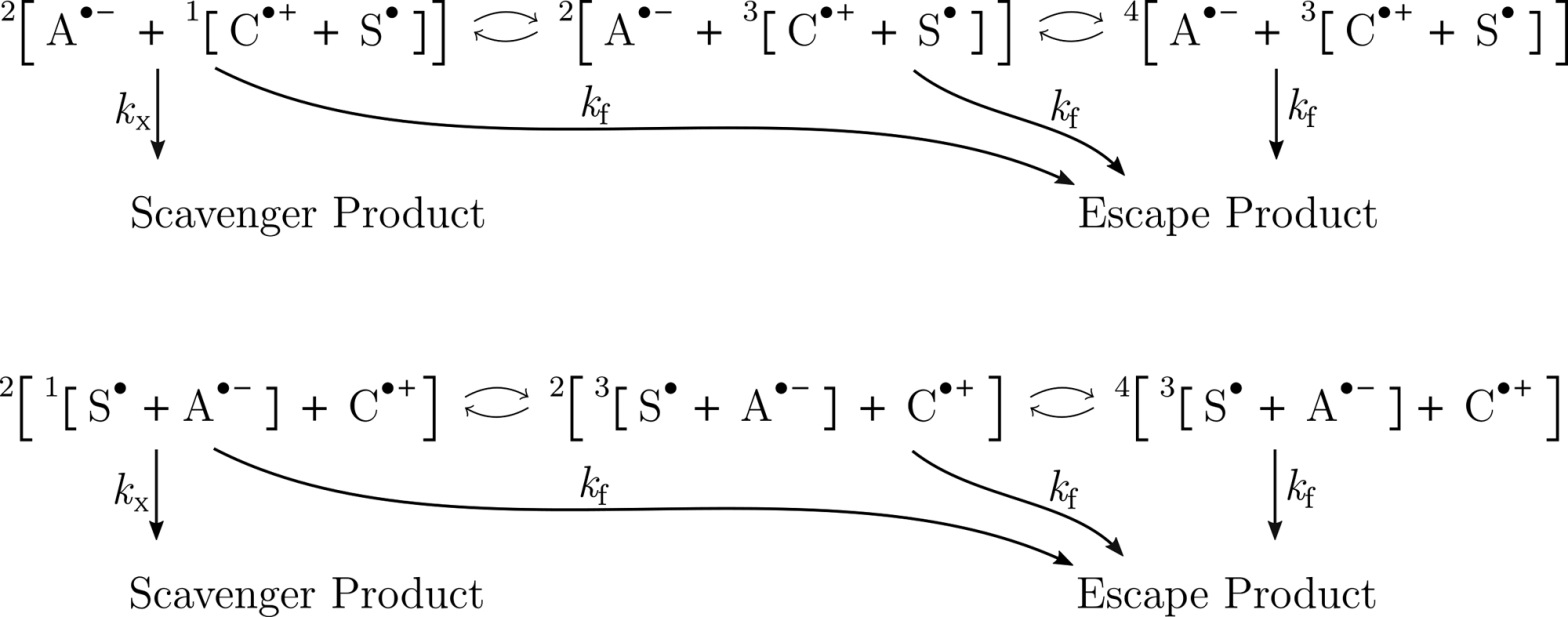
Three-Radical Mechanism, Wherein Scavenger S^•^ Reacts with Cation C^•+^ (Top) or A^•–^ (Bottom) of an Anion/Cation Radical Pair [A^•–^/C^•+^] The rate constants indicate scavenging (*k*_X_) or escape (*k*_f_) and paired arrows indicate coherent interconversion processes. The primary recombination of the [A^•–^/C^•+^] radical pair (feasible from the overall doublet spin state) has been disregarded, as it has only a minor impact on the reaction anisotropy if *k*_X_ is large.

Here, we assesses the prospect of the scavenging reactions Scheme 3 to identify a feasible design principle for a biological magnetoreceptor. We are careful to account for EED couplings, demonstrating how radical scavenging may sustain large magnetosensitivity in their (unavoidable) presence. The following Theory section introduces the spin-physical chemistry, and its schematic representation. In the Model section, we introduce radical scavenging into prototypical cryptochrome systems to explore prospects for scavenger-mediated magnetoreception. Among our Results, we demonstrate the feasibility of a magnetosensitive chemical pathway in cryptochrome based on three-radical correlations in model systems, where one member of the primary radical pair reacts with a scavenger radical, in contrast with the predictions of comparable RPM or R3M models. We summarize with a Discussion of our findings, and draw Conclusions in light of this new prospect for radical magnetosensation.

## Theory

We formulate the model in the context of a generic anion–cation radical pair [A^•–^/C^•+^], complimented by a radical scavenger S^•^ that reacts with one of the two primary-pair radicals to yield a distinct diamagnetic product (Scheme 3). In practice, we consider three-radical systems comprising S^•^ and one of the two established primary radical pairs, either [FAD^•–^/W^•+^] or [FADH^•^/Z^•–^]. The coherent spin dynamics in this system are governed by the respective Zeeman, EED, hyperfine, and exchange effects, according to the Hamiltonian *Ĥ* = *Ĥ*_Zee_ + *Ĥ*_EED_+ *Ĥ*_hf_ + *Ĥ*_ex_, as detailed in the Supporting Information (SI). The EED coupling was modeled based on the point-dipole model, whereby all pairwise interactions of the three radicals were considered explicitly. Reaction channels consist of the forward reaction (rate *k*_f_) and scavenging (rate *k*_X_). The term “chemical Zeno Effect” describes the reaction dynamics enabled by this scheme.^65^ Specifically, the scavenging reaction converts triplets to singlet states of the original pair, even in the absence of coherent interaction terms, and thus resembles the quantum (anti-)Zeno effect.^66^ The directionality of the MFEs, here, results from the combined action of the anisotropic hyperfine and EED coupling, mediated by the incoherent recombination dynamics.

To simulate the anisotropic MFEs in the presence of EED coupling and radical scavenging, we numerically integrated the Liouville–von Neumann equation describing the spin dynamics of the three-radical state of the system:
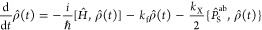
1where ρ̂(*t*) represents the time-dependent spin-density matrix of the activated complex, and the Hamiltonian *Ĥ* comprises all Zeeman, EED, hyperfine, and exchange effects (see SI). The operator *P̂*_S_^ab^ projects onto the singlet state of the two radicals labeled *a* and *b*. For the scavenging reaction, that is, according to the reaction Scheme 3, we have (*a*,*b*) = (1,3) or (2,3) where labels 1 and 2 indicate the primary pair radicals, and where 3 designates the scavenger radical. The brackets [] or {} denote commutators or anticommutators, respectively.

In the case of the [FAD^•–^/W^•+^] primary radical pair, the geminate pair is assumed to be “born” as a singlet ρ̂(0) ∝ *P̂*_S_^12^. Whereas in the case of the [FADH^•^/Z^•–^] primary pair, it is born as a triplet ρ̂(0) ∝ *P̂*_T_^12^ (where  is the triplet projector). In either case, the scavenger is initially uncorrelated with the primary radical pair. We emphasize that we have purposefully neglected primary-pair recombination from models of primary-pair scavenging here, to assess the viability of magnetoreception via radical scavenging on its own. Previous investigations indicated that primary-pair recombination has only a minor effect on the MFE when a scavenging reaction dominates the spin dynamics.^63^

The formulation of this modeling framework enabled us to predict the forward reaction yield Φ_f_ = *k*_f_ ∫_0_^∞^ Tr[ρ̂(*t*)] d*t*, and the scavenging yield Φ_X_ = *k*_X_ ∫_0_^∞^ Tr[*P̂*_S_^*a*3^ ρ̂(*t*)] *dt*, for *a* ∈ {1,2}. Assuming a magnetic flux density of 50 μT to represent the geomagnetic field, we simulated the reaction yield for 2562 distinct field orientations (i.e., 1281 axes), to estimate the maximum Γ (over orientations) of the relative MFE anisotropy in each scenario
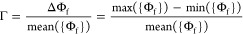
2where the yield maximum (max), minimum (min), and mean in eq 2 are evaluated over the 1281 magnetic field axes considered.

## Models

To show how the chemical Zeno effect may generate magnetosensitive spin dynamics in cryptochrome in the presence of EED coupling, we modeled systems of three radicals, based on structural models of cryptochrome determined for animal species *Columba livia* (*Cl*Cry4, PDB 6PU0) and *Drosophila melanogaster* (*Dm*Cry, PDB 4GU5).^57,67^ For each protein, we considered the two primary radical pairs [FAD^•–^/W^•+^] and [FADH^•^/Z^•–^]. For each pair, we simulated one of two possible geometric arrangements, one with the primary-pair radicals spaced relatively close together (i.e., [FAD^•–^/W_C_^•+^] or [FADH^•^/Z_near_^•^]), and another with them spaced far apart (i.e., [FAD^•–^/W_D_^•+^] or [FADH^•^/Z_far_^•^]). The more distant spacings were chosen to satisfy the need to minimize detrimental EED interactions a priori, thus realizing conditions favorable for the spin dynamics of the RPM. Respectively, W_318_ and W_369_ correspond to W_C_ and W_D_ of *Cl*Cry4. Likewise, W_342_ and W_394_ correspond to W_C_ and W_D_ of *Dm*Cry. The Z_near_^•–^ and Z_far_^•–^ positions imply a radical that is either tucked within 5 Å of the FAD cofactor, or placed at a distance about 18 Å away from the FAD (as in Figure 1; see also Figure S1 in the SI). These two Z^•–^ locations were introduced in prior work.^55^

The relative coordinates of all reaction partners are reported in the SI, along with the hyperfine parameters used and other pertinent details. For the Z^•–^-containing systems, we consider the neutral flavin semiquinone FADH^•^ in the assumption that this radical pair is produced from the fully reduced FADH^–^ by oxidation; for the W^•+^-containing radical pairs implicated with the photoreduction we assume the anion radical, FAD^•–^, that is, the direct product of the electron transfer to the FAD resting state, the protonation of which proceeds on a time scale slow compared to the magnetic field-dependent spin dynamics.^41^ As *Dm*Cry does not undergo complete photoreduction (but can still be chemically reduced), we will additionally consider the alternative [FAD^•–^/Z^•–^] radical pair.^68^ We note that members of the cryptochrome family in general exhibit homology in their three-dimensional fold and conservation of critical amino acids. While the crystal structure of the European Robin cryptochrome 4 (*Er*Cry4), recently highlighted as a magnetoreceptor in ref (44), has not been resolved, the homology model from ref (25) shows comparable spatial orientation of its FAD, W_C_ and W_D_ residues (see Figure S2). Thus, for the rate constants assumed here (those found in ref (44) do not support substantial magnetosensitivity in the geomagnetic field), comparable MFEs are expected for this arguably more relevant, but structurally less well-defined protein. We, furthermore, point out that *Cl*Cry4 crystal structure is lacking part of the phosphate-binding loop. Here, *Dm*Cry can serve as a template representing a generic cryptochrome structure, even if its photocycle might differ. In the Results section, we will therefore describe auspicious scavenger locations with respect to the *Dm*Cry structure, as these can easily be remapped onto homologous cryptochromes.

We studied numerous three-radical scenarios, using the four radical pairs described above as starting points. We represent these simulation scenarios by designating the three radicals and using parentheses to indicate the reactive pair. For example, (S^•^/FAD^•–^)/W^•+^ stands for the singlet-born [FAD^•–^/W^•+^] scenario wherein the FAD^•–^ is scavenged by radical S^•^, whereas FADH^•^/(Z^•–^/S^•^) designates the triplet-born [FADH^•^/Z^•–^] with Z^•–^ susceptible to scavenging. See Table 1 and Table S1 for a comprehensive list of the systems studied.

**Table 1:** Maximum Relative MFEs, Γ_max_ = max_*R*_ Γ_β_(*R*), with the Corresponding Optimal Scavenger-Target Distances *R*_max_^a^

	β = 4.0 Å^–1^		β = 2.8 Å^–1^		β = 1.4 Å^–1^		β = 0.9 Å^–1^	
radical scavenger mechanism	Γ_max_ (%)	*R*_max_ (Å)	Γ_max_ (%)	*R*_max_ (Å)	Γ_max_ (%)	*R*_max_ (Å)	Γ_max_ (%)	*R*_max_ (Å)
FAD^•–^/(W_318_^•+^/S^•^)	17.3	5.7	14.9	6.8	14.7	10.0	29.3	15.9
FAD^•–^/(W_369_^•+^/S^•^)	27.4	5.7	36.6	6.8	24.1	9.9	44.7	16.2
(S^•^/FAD^•–^)/W_318_^•+^	6.9	5.1	8.3	6.6	7.7	11.2	23.4	16.8
(S^•^/FAD^•–^)/W_369_^•+^	9.7	5.2	7.6	6.0	9.3	11.1	28.7	16.8
FAD^•–^/(W_342_^•+^/S^•^)	17.9	5.7	16.5	6.6	16.8	11.0	32.6	16.1
FAD^•–^/(W_394_^•+^/S^•^)	30.0	5.7	36.5	6.8	24.6	9.9	43.9	16.2
(S^•^/FAD^•–^)/W_342_^•+^	7.3	5.1	8.2	6.4	7.7	11.2	23.5	16.5
(S^•^/FAD^•–^)/W_394_^•+^	10.5	5.2	10.4	6.0	8.0	11.0	29.0	16.8
FADH^•^/(Z_near_^•–^/S^•^)	38.5	5.6	29.3	5.6	29.4	10.3	36.3	15.1
FADH^•^/(Z_far_^•–^/S^•^)	4.8	6.0	13.7	7.0	36.8	9.8	53.8	15.0
(S^•^/FADH^•^)/Z_near_^•–^	73.9	4.9	68.3	5.3	24.9	8.1	25.4	15.8
(S^•^/FADH^•^)/Z_far_^•–^	30.8	5.7	32.3	6.7	22.9	9.9	30.0	15.7

a Respectively, W_318_ and W_369_ correspond to W_C_ and W_D_ of *Cl*Cry4. Likewise, W_342_ and W_394_ correspond to W_C_ and W_D_ of *Dm*Cry. The use of brackets indicate the radicals involved in the scavenging process (cf., Table S1).

Initially aiming to estimate the largest anisotropic MFEs that might be enabled by a scavenger radical introduced to combine with one member of a radical pair, we performed an unconstrained MFE optimization by independently varying the scavenger location and the scavenging rate for the (S^•^/FAD^•–^)/W_D_^•+^ reaction in *Dm*Cry (see SI, Figure S3). Applied to the other systems under investigation, the fit to this preliminary optimization generated tremendous predictions of Γ exceeding 100% for distantly removed scavenger radicals, while implicating inordinately large scavenging rates (relative to the distances from cryptochrome involved) in all of the systems under consideration. For example, the (S^•^/FAD^•–^)/W_C_^•+^ model of *Cl*Cry4 yielded a relative anisotropy of 158% for a distance of 100 Å from FAD^•–^ to S^•^ and a scavenging rate *k*_X_ = 3 × 10^7^ s^–1^. For FADH^•^/(Z_far_^•–^/S^•^), we obtained Γ = 147% for a scavenger at a distance of 45 Å from Z_far_^•–^ and a rate *k*_X_ = 3 × 10^7^ s^–1^. These naïve predictions are summarized in Table S2, and presented in detail in Figures S3–S6. What they share in common is that they require rate constants that exceed even the most optimistic predictions of charge-transfer rates afforded by nonadiabatic ET theory (e.g., activationless ET along a covalently linked bridge).^69^ These outlandish estimates revealed a need to bound our procedure to exclude inordinate ET rates and unrealistically large MFEs.

To achieve this, we, subsequently, incorporated considerations of long-range ET into our scavenger-based cryptochrome compass model. For long-range biological ET,^69^ the charge transfer may be modeled using a one-step nonadiabatic process defined by Marcus theory.^70^ The Marcus ET rate is limited by the exponential decay of the coupling matrix element with the donor–acceptor separation. This allowed us to constrain our simulation procedure, restricting scavenging rates to those bound by Marcus’ equation^71^ by assuming ET to be activationless and limited by the (first-order) dynamics of electron tunneling through the biological medium. We considered the fastest possible ET rates to allow for the maximum possible reactant distances, because the spin-chemistry of the RPM model is governed by competition between the need to reduce EED coupling while sustaining a large enough charge-combination rate to produce a significant reaction-yield MFE.^55^

To this effect, we bounded charge combination rates from above by assuming scavenging by activationless ET according to the “Moser–Dutton” (MD) rate equation:^72^
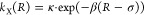
3where *R* is again the distance from the scavenger (S^•^) to its redox target, σ = 3.6 Å is the minimal distance of the redox partners (i.e., a typical van der Waals distance), and β is the decay parameter associated with the square of the ET coupling matrix element. The tunneling parameter β is expected to vary from 0.9 to 4.0 Å^–1^, depending on intervening matter,^69,73^ and κ = 10^13^ s^–1^ reflects a typical rate for reactants in van der Waals contact.^74^

For all our simulations, we assumed the primary pair to “escape” to the signaling state on the time scale *k*_f_^–1^ = 3 μs, consistent with estimates of the primary pair’s lifetime.^40,41,75^ To choose β, we examined four cases: ET between one primary radical and the scavenger via a covalent bridge (β = 0.9 Å^–1^), a typical protein (β = 1.4 Å^–1^), a weakly coupled medium (β = 2.8 Å^–1^), or an extremely weakly coupled (vacuum-like) system (β = 4.0 Å^–1^).^69,73,76^ This range of β-values is comparable to that used in a previous study on EED effects on radical pair recombination.^54^ The strong coupling limit (β = 0.9 Å^–1^) provides a fixed upper bound on the activationless ET rate and, thus, provides an appropriate maximum-coupling limit from which to model biochemical MFEs under increasingly rate-limited circumstances. For the sake of simplicity, we have presented all rate limiting effects in the form of the single parameter model eq 3 based on the effective decay parameter β. Clearly, the choice of β = 0.9 Å^–1^, typically quoted for covalent bridges, reflects a best-case scenario insofar as it allows placing the radicals maximally apart. While optimistic in view of the protein environment relevant here, low beta-values are not uncommon in biological context: β = 1.1 Å^–1^ has been reported for the distance dependence of driving-force-optimized ET rate constants of Ru-modified proteins and the pathway model suggest β = 1.0 Å^–1^ for coupling along β-strands.^77^ Further note that protein ET rate data always show substantial scatter reflecting the important features of the protein medium in terms of bond and hydrogen bond structure, and often realizing significantly stronger coupling than reflected by the average β.^69^ In fact, long-distance interprotein electron transfer reactions (e.g., between cytochrome c and the mitochondrial complex III) proceeding through aqueous solution with extraordinary β = 0.15 Å^–1^ over distances of up to 10 nm have been described.^78^ Incoherent hopping through real redox intermediates at moderate driving force and dynamically limited adiabatic ET are also associated with slow distance–decay.^77^

Likewise, we wish to emphasize that the rates chosen to reflect the most severely rate-limited ET may effectively (or even preferably) be viewed as those of protein-coupled ET across a non-zero-free-energy barrier. In this way, our one-parameter ET decay model enables sampling of significant regions of the reaction configuration space by accounting for distance-dependent coupling, needed to inform inferences into the nature of scavenging reactions for biochemical magnetic sensing. While more complete models can and should be employed in subsequent studies, the large number of parameters in such models would make a systematic exploration of multiple reaction parameters in the current study more computationally costly, without actually affecting the range of possible scavenging rates under consideration here (see below). Finally note that large β-rates can also result from multipathways scenarios because of the destructive interference intrinsic in competing coupling routes^79^ and might offer a better description for radical scavenging reactions associated with the formation of covalent bonds (e.g., consider the ascorbyl radical and the flavin semiquinone reacting with superoxide under formation of hydroperoxides), which are expected to proceed at contact.

## Results

We performed MFE simulations, systematically sampling scavenger S positions around each primary radical pair (within 3 nm of each radical target, FAD, W, or Z). To constrain the scavenging ET rate *k*_X_ (at a given radial distance *R* from the scavenger from its target) to reasonable values, we introduced eq 3 in combination with select ET decay constants β. As our top “speed limit,” we elected to use a model of activationless charge transfer via optimal tunneling through the covalent bonds of an ideal β sheet.^73,76^ This limit reflects the largest plausible rate of nonadiabatic ET through an organic bridge, thus providing an effective bound on the distance from the scavenger from its target in the rich biological *milieu*. This is distinct from the MFE itself, which also depends on the scavenger position as a consequence of including both the distance *and* orientation dependence of the EED coupling (assuming point dipoles).

To account for less-than-ideal ET, we also considered larger decay constants β > 0.9 Å^–1^, including one typical of coupling in a well-optimized biological environment β = 1.4 Å^–1^, and two weaker couplings β = 2.8, 4.0 Å^–1^ (which could also designate protein-mediated ET over a modest activation barrier, considered below). This allowed us to explore realistic ET rates spanning a wide range of rates. Therefore, we evaluated a maximum relative MFE anisotropy Γ as a function Γ_β_(*R*) of *R* for each scavenged primary pair for each decay parameter β. This afforded us a total of 48 distinct scenarios by considering each of two model Cry structures (*Dm*Cry and *Cl*Cry4), two primary radical-pair types ([FAD^•–^/W^•+^] or [FADH^•^/Z^•–^]), two distinct scavenger targets (either FAD or its primary partner), and four values of the decay constant β—where there is no difference between results obtained from using different Cry structures for the alternative [FADH^•^/Z^•–^] primary radical pair. These 48 mechanisms are designated in the 48 distinct Γ_max_ entries listed for the constrained ET in Table 1.

Table 1 predicts the existence of *optimal* and *robust* scavenger configurations: These are “optimal” insofar as they predict the largest maxima (given constraints), and “robust” in that they predict *consistently* large maxima across the values of β considered. For both *Cl*Cry4 and *Dm*Cry structures, the FAD^•–^/(W_D_^•+^/S^•^) models predict large maximum MFE anisotropies Γ_max_ = max_*R*_ Γ_β_(*R*) > 20% for *all* of the decay parameters explored. Likewise, the FAD^•–^/(W_C_^•+^/S^•^) models consistently predicted MFE anisotropies Γ_max_ ≥ 15%. The bird cryptochrome did not predict larger MFEs than those of the fly in the cases studied. The FADH^•^/(Z_near_^•–^/S^•^) models predicted large and robust MFEs, with global maxima near 30% (or even 40%) for all β considered. These provide contrast to the predictions of the FADH^•^/(Z_far_^•–^/S^•^) model, for which the MFE maxima were not particularly large nor robust with respect to variations in β, increasing from the modest Γ_max_ ≈ 5% (for β = 4.0 Å^–1^) to large values Γ_max_ > 35% for activationless protein-mediated ET between Z^•–^ to a distant S^•^. The optimal distance is determined by the value of β and is only weakly dependent on the identity of the primary radical pair. For the systems studied in Table 1 and each decay coefficient β ∈ {4.0, 2.8, 1.4, 0.9} Å^–1^, we obtained the mean optimum distances *R*_max_ ∈ {5.5, 6.3, 10.2, 16} Å, respectively, such that *k*_X_ ∈ {5.0, 5.2, 1.1, 0.1} ns^–1^. This indicates that a shorter distance between the scavenger and its target (due to faster decay of the coupling matrix element) may be compensated by a faster reaction with the scavenger radical. Regardless of the ET model employed, scavenging mechanism could enable large Γ_β_(*R*) for some plausible choice of (β,*R*). This is unlike the RPM, for which EED coupling is not counteracted by increasing the charge transfer rate.

**Figure 2:**
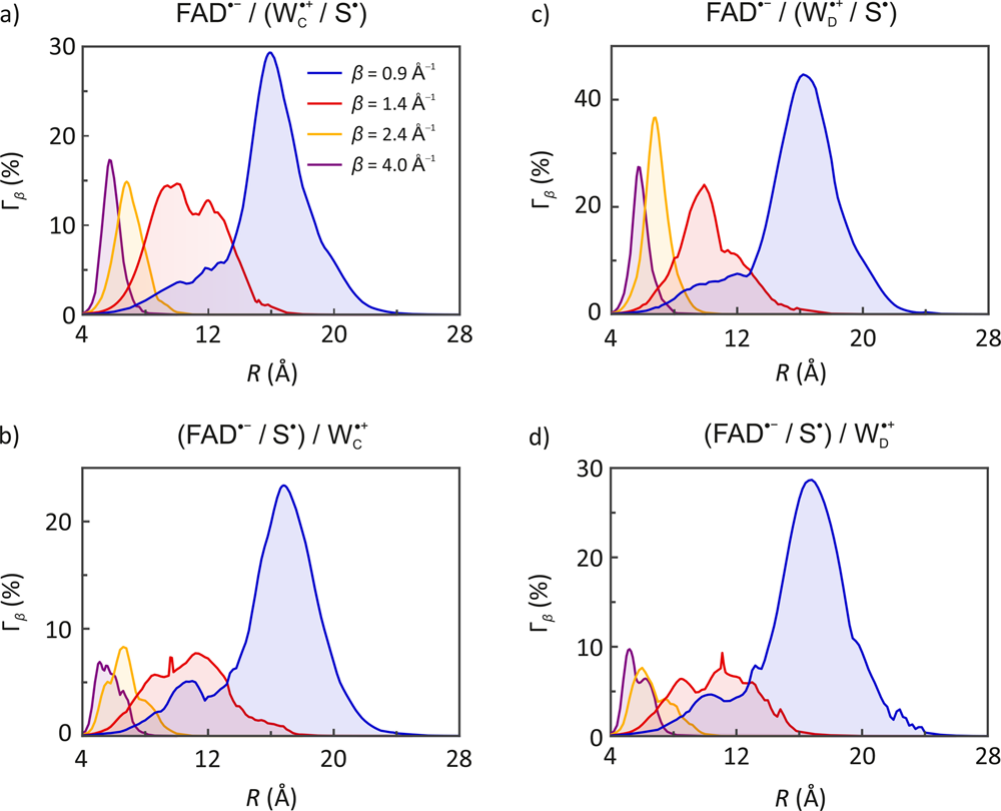
Maximum relative MFE anisotropy Γ_β_(*R*) by distance to the scavenger S^•^, for models with a FAD^•–^/W^•+^ primary pair in *Cl*Cry4, based on activationless ET through four tunneling media: covalently bound (blue), typical protein (red), “soft” vacuum (yellow), and “hard” vacuum (purple). Panels a and c show MFEs from simulations of W^•+^ scavenged by S^•^, whereas panels b and d show results for FAD^•–^ scavenged (see Scheme 3). Tunneling decay parameters are indicated in the legend of panel a, which applies throughout. Brackets are used in the panel labels to indicate the radical being scavenged.

**Figure 3:**
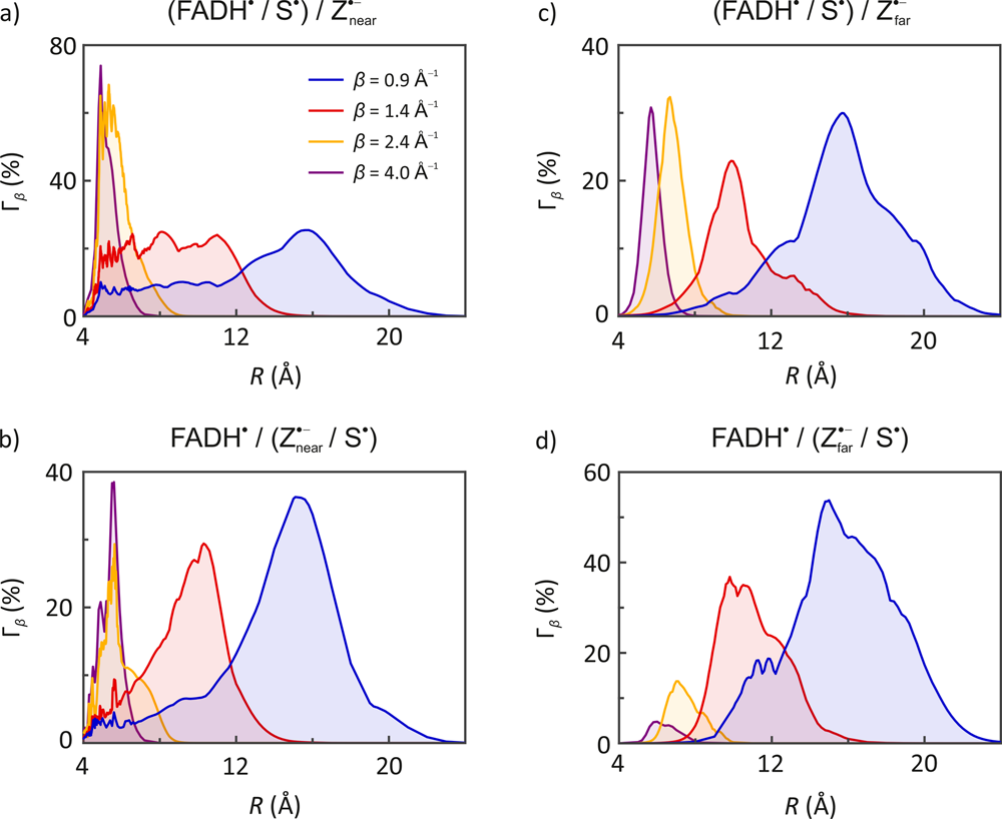
Depicts maximum relative MFE Γ_β_(*R*) by distance *R* from scavenger S^•^ for each FADH^•^/Z^•–^ model, based on activationless ET through four tunneling media: covalently coupled (blue), typical protein (red), “soft” vacuum (yellow), and “hard” vacuum (purple). Panels a and c show results from simulations of FADH^•–^ scavenged by S^•^, whereas panels b and d show MFEs from simulations of Z^•–^ scavenged by S^•^; see Scheme 3. Tunneling decay parameters are shown in the legend of panel a. Brackets are used in the subfigure labels to indicate which radical is being scavenged (cf., Table S1).

**Figure 4:**
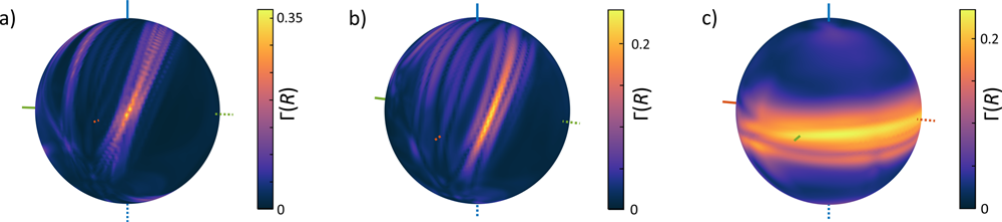
Dependence of the relative anisotropy Γ on the location of the scavenger radical at a given distance *R* from its reaction partner is depicted for three scenarios: (a) FAD^•–^/W_D_^•+^ radical pair in *Dm*Cry with W_D_^•+^ scavenged by radical S^•^, β = 2.8 Å^–1^, and *R* = 6.8 Å; (b) FAD^•–^/W_D_^•+^ radical pair in *Cl*Cry4 with W_D_^•+^ scavenged by radical S^•^, β = 1.4 Å^–1^, and *R* = 9.9 Å; and (c) FAD^•–^/W_C_^•+^ radical pair in *Dm*Cry with FAD^•–^ scavenged by radical S^•^, β = 0.9 Å^–1^, and *R* = 17 Å.

Figures 2 and 3 show the maximum MFEs predicted at scavenger distance *R* from the radical being scavenged for *Cl*Cry4 for each coefficient β ∈ {0.9, 1.4, 2.8, 4.0} Å^–1^ (see Figures S10 and S11 for plots of the associated *ΔΦ*_f_ and Figure S8 for results on *Dm*Cry). For covalently bridged ET (β = 0.9 Å^–1^), the peaks in these curves correspond to fairly broad maxima (blue curves, Figures 2 and 3), rather than a narrow optimum indicating a strictly defined preferred location (also compare Figure 4a, b and c). Simulations of scavenging rates typical of biological ET (β = 1.4 Å^–1^) predicted tightly localized MFE maxima that could indicate preferential scavenger positions (Figure 4 b). In the scavenged [FADH^•^/Z^•–^] models, the scavenging of FADH^•^ produced particularly large anisotropies for weak coupling over a short scavenger-target distance, again implicating sharp maxima. It is remarkable that such large sensitivity can ensue despite the small radical distances, as the EED coupling becomes the dominant interaction in the spin Hamiltonian (with interaction parameters of the order of hundreds of MHz, which entirely abolishes MFEs in the RPM).

The (S^•^/FADH^•^)/Z_near_^•–^ model predicted Γ_max_ ≈ 70% using a scavenger radical placed about 5 Å away from Z_near_^•^ for each of the vacuum-like decay parameters (i.e., β = 2.8 or 4.0 Å^–1^). Those results indicated a substantial prospect for directional MFEs generated by an FADH^•^-scavenged reaction in the presence of a Z^•–^*bystander* (initially formed as part of the original [FADH^•^/Z^•–^] pair), regardless of the position of Z^•–^ in space *or* the scavenger’s reactivity with FADH^•^. Furthermore, both the *Dm*Cry and *Cl*Cry4 models gave similar results, with the fly (*Dm*Cry) cryptochrome performing marginally better than the that of the pigeon (despite a larger primary–pair separation) in this setting.

Overall, we found that all of the scavenger models based on Scheme 3 predicted greatly improved MFEs, as opposed to the RPM-based mechanisms shown in Scheme 1, with maximum anisotropies ranging between 5% and 75% (Table 1). It is worthwhile to draw attention to the fact that largest MFEs were predicted by the most weakly coupled models (i.e., with large β) electron tunneling. This is significant because those same ET rates could equivalently implicate protein- or solvent-mediated ET across a small activation barrier. For example, eq 3 gives the ET rate over a distance of 7 Å with β = 2.8 Å^–1^ to be *k*_X_ = 0.7 ns^–1^, which is identical with the rate of ET over the same distance through typical protein (β = 1.4 Å^–1^) with an activation barrier of 4.8 *k*_B_*T* (viz., Figure 4a). Likewise, activationless ET over a distance of 6 Å with effective β = 4.0 Å^–1^ gives rate of *k*_X_ = 0.7 ns^–1^, which is the same as that of ET across the same 6 Å distance through typical protein (β = 1.4 Å^–1^) when assuming an activation barrier of 6.2 *k*_B_*T*. This presents the possibility that ET may be optimized with respect to the ET parameters describing the solvent–protein structure. Importantly, large MFEs such as the 35% MFE shown in Figure 4a (with an effective decay parameter of β = 2.8 Å^–1^) could be achieved for an activated ET processes with actual decay parameter of β = 1.4 Å^–1^ typical of protein.

To explore the scavenger’s dependence on β for each reaction type, we have considered Γ_β_(*R*) by calculating the maximum MFE over all locations on a sphere of radius *R*. Figure 2 shows the dependence of Γ_β_(*R*) on the distance *R* from the scavenger S^•^ to its target, for reaction models initialized with the conventional [FAD^•–^/W^•+^] pair. Figure 3 shows these profiles for scavenger-reaction models derived from the alternative [FADH^•^/Z^•–^] pair. As expected, these diagrams reflect the trends discussed above. For small β in particular, the profiles are wider with maxima occurring at large distances; whereas for large β they are peaked at small distances. We further used these profiles to systematically search for optimal scavenger locations by recursively increasing the number of sampling points at a given *R*.

Predictions of Γ_max_ ≥ 20% for a scavenger located near Trp (or Tyr) residues in the cryptochrome structures are particularly significant here. In *Dm*Cry, we found that a W_D_^•+^ scavenger, positioned in the vicinity of the residue Met506, provided MFEs of about 20%, which were robust with respect to variations in the tunneling decay parameter between 1.4 Å^–1^ ≤ β ≤ 4.0 Å^–1^. Likewise, a Z_near_^•–^ scavenger, located near Trp420 (i.e., W_A_) produced significant MFEs of about 10% over the same tunneling decay range, consistent with the position of a scavenger implicated near W_A_ in *Cl*Cry4. Simulations of protein-mediated ET (β = 1.4 Å^–1^) predicted W_D_-scavenged Γ_max_ > 20% (or W_C_-scavenged Γ_max_ > 10%) for scavengers in close-contact with a Tyr residue near the end of the Trp-tetrad in *Cl*Cry4.

The predictions of the (S^•^/FADH^•^)/Z_near_^•–^ model corresponded to Γ_max_ > 70% for a scavenger radical placed about 5 Å away from both Z_near_^•^ and FADH^•^ for β = 4.0 Å^–1^, or Γ_max_ > 65% for a pair of maxima within 6 Å of Z_near_^•–^ and FADH^•^ for β = 2.8 Å^–1^ (but in opposite directions). The FADH^•^/(Z_near_^•–^/S^•^) model predicted Γ_max_ > 37% for a scavenger nestled near the FAD flavin and Asp387 of *Cl*Cry4. The (S^•^/FADH^•^)/Z_far_^•–^ model predicted Γ_max_ ≈ 30% for scavenger radicals nestled near to the FAD flavin, in close contact with Ile390 for both β = 2.8 and β = 4.0 Å^–1^. Finally, the FADH^•^/(Z_far_^•–^/S^•^) model predicted Γ_max_ ≈ 35% for scavengers with β = 1.4 Å^–1^, placed either in contact with the FAD phosphate or near the protein surface about 5 Å away from both His352 and His354.

As the triplet-born FADH^•^/Z^•^ implicates a reoxidation reaction from the fully reduced FADH^–^, which is not accessible by photoreduction in *Dm*Cry (but could still be formed by chemical reduction),^68^ we have furthermore tested the alternative model of a scavenger FAD^•–^/Z^•–^ radical pair born with random spin configuration (i.e., as F-pair). Such a pair could conceivably result in *Dm*Cry after the initial photoreduction by encounter with a O_2_^•–^. As shown in Figure S9, the magnetosensnitivity of this model even exceeds that of the scavenger FADH^•^/Z^•^, demonstrating general applicability of the model even for initial states that are not spin-correlated.

**Figure 5:**
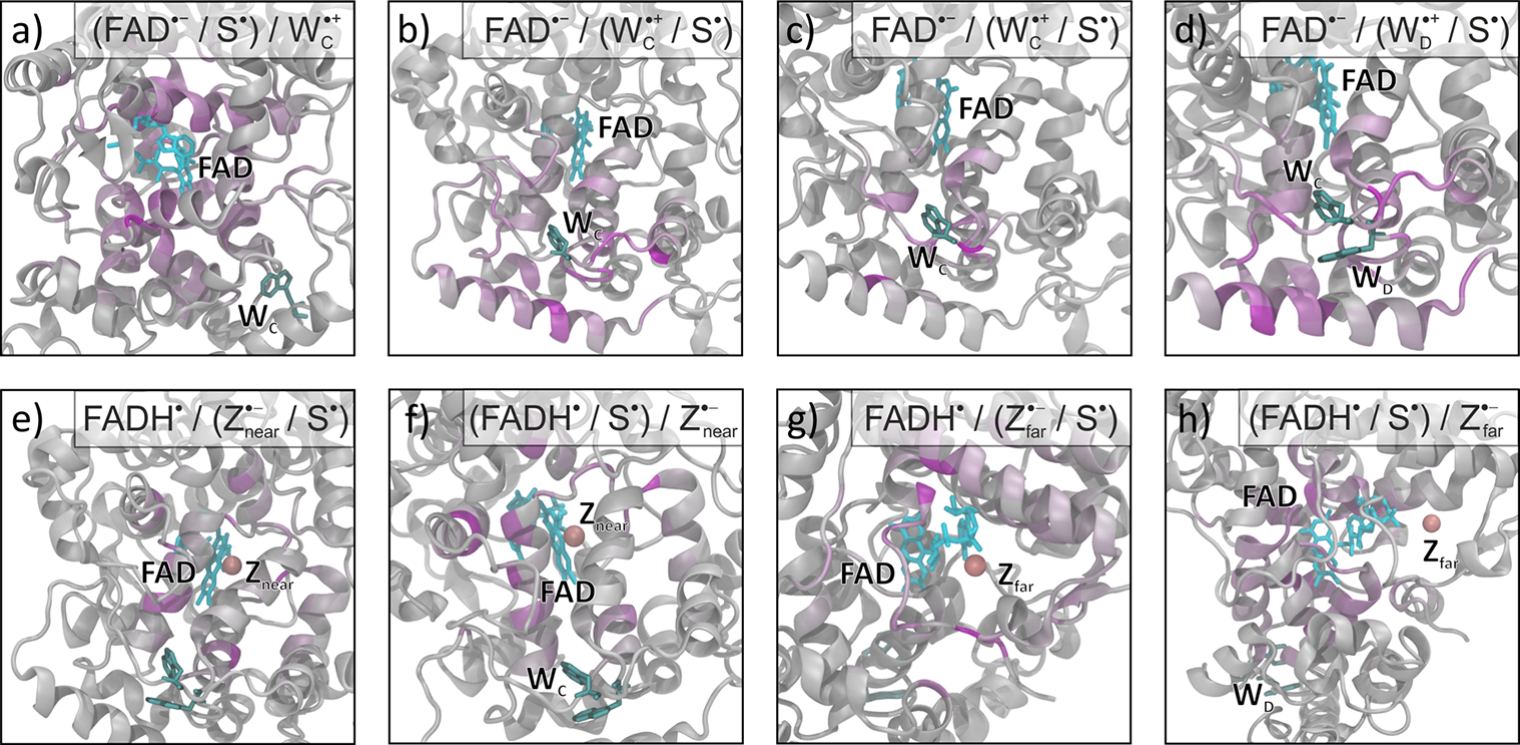
Protein structures of the *Dm*Cry color-coded by the maximal relative anisotropy realized by placing the scavenger at the location of individual protein residues, that is, formally identifying the scavenger with the residue. For all panels, except panel c, β = 1.4 Å^–1^; for panel c, β = 2.4 Å^–1^. The scenarios considered are indicated in the panels.

Figure 5 provides an overview of the efficiency of scavengers placed within the protein. To this end, each protein residue of *Dm*Cry was colored by the maximal relative MFE elicited at the location of its heavy atoms. *Dm*Cry was used here as a template as its crystal structure is complete in the surroundings of FAD. In any case, note that for many scenarios the maximal relative anisotropy is realized outside of the protein envelope. Note, furthermore, that if β is increased in these plots, the optimal sites move closer to the reaction partner’s location, as expected, while the optimal directions are roughly preserved (cf., Figure 5b and c). A more detailed summary of optimal scavenger locations is given in the SI.

To provide a direct comparison of the scavenged radical-pair model to the bystander-enhanced model (R3M) and the standard RPM under equivalent conditions, we estimated equivalent recombination-based MFEs using the same ET decay coefficients (β), relative radical locations, hyperfine, and dipolar interactions. To do so, we considered an inert “bystander” radical B^•^ taking the place of S^•^, re-engaged the primary radical pair recombination and carried out a further set of simulations. Using recombination rates derived from eq 3 with the same decay factors β ∈ {0.9, 1.4, 2.8, 4.0 } Å^–1^. We systematically explored bystander positions on spheres of radii *R*_13_, centered around the FAD cofactor, thus recovering the standard RPM scheme in the limit of very large FAD-scavenger distance *R*_13_.^55^ These results may be found in Figure S7.

To wit, the R3M models based on recombination between FAD^•–^/W^•+^ pairs afforded MFEs of at most 1% (for FAD^•–^/ W_C_^•+^) or 2% (for FAD^•–^/W_D_^•+^), and only in the activationless limit of recombination by assuming ET directly across a covalent bridge linking FAD to TrpH in the presence of a bystander radical B^•^. That assumption *could* be tolerable for the FAD^•–^/W_C_^•+^ pair with radical lifetime between 3 and 10 μs,^40,75^ which are comparable to that of long-range ET over the same distance with a well-optimized tunneling coefficient (i.e., β between 1.2 and 1.3 Å^–1^). However, the lifetime of the more distantly separated FAD^•–^/W_D_^•+^ pair would correspond to a value of β ≈ 1.4 Å^–1^, typical of protein-mediated ET (cf., Figure S7b). Hence, the direct recombination of W_D_^•+^ with FAD can scarcely provide a viable mechanism in Cry, either for the R3M (with a bystander nearby), or for the RPM model alone (i.e., with bystander removed) where MFEs < 0.1% because of the suppressive EED interaction. As its lifetime is 3 orders of magnitude longer than that of the W_C_^•+^-based counterpart, it would furthermore be strongly attenuated by spin relaxation, an effect which has not been taken into account here.

Similarly, the R3M-based models of FADH^•–^/Z_far_^•–^ magnetoreception predicted MFEs no larger 4%, and then again only in the scenario with primary pair recombination governed by covalently bridge ET (β = 0.9 Å^–1^) and with a catalytic bystander nearby. The RPM alone, again predicted MFEs of no more than about 0.1% even assuming fast ET, again because of the suppressing effect of the EED interaction. On the other hand, models of recombination in the FADH^•–^/Z_near_^•–^ primary pair predicted sizable MFEs approaching 8% with a bystander radical nearby (i.e., within 5 Å of the FAD, assuming vacuum-mediated back-ET). This reserves the possibility for the R3M-based [FADH^•–^/Z_near_^•–^] sensory system, where the B^•^ sits next to the flavin cofactor (see SI)—contingent on the existence of a slow-relaxing Z^•–^.

## Discussion

The absence of a robust candidate model has left a gap between conflicting claims in the chemical magnetoreception literature.^20,23,30,51^ A proof-of-principle of a RPM-based compass was engineered and synthesized using a covalently bridged radical-pair system in a laboratory,^80^ but the compelling demonstration of a biological radical-pair reaction, sensitive to the direction of an Earth-strength (50 μT) field, remains to be shown beyond circumstantial evidence. The demonstration of a practical cryptochrome geocompass would provide strong support for the magnetoreceptor hypothesis, but its absence raises the question: Does the RPM provide an appropriate model of cryptochrome-mediated magnetoreception?

The radical-scavenging mechanism proposed in this work as a model chemical compass stands apart from the traditional RPM-based schemes, by allowing one member of the radical pair to be either taken up by a dedicated scavenger (to a nonsignaling product) or to escape to the signaling state. To illustrate the distinction between this scavenger-based model and recombination-based ones (such as the RPM or R3M), here, we have assumed that the primary radical pair does *not* recombine. Therefore, radical pair recombination is not a prerequisite for chemical magnetoreception, although it is equally not excluded (i.e., robust scavenger-based MFEs in the presence of primary-pair recombination are still expected).^63^ Whereas the extension of the RPM by a third bystander radical (i.e., the R3M) can mitigate the reduction in magnetic sensitivity because of the presence of unavoidable EED coupling,^55^ the scavenging mechanisms circumvents certain otherwise-insurmountable problems. In particular, we have shown that the scavenger-based mechanism predicts large directional magnetosensitivity in the presence of EED coupling (needed for chemical magnetoreception), even when the ET coupling to the scavenger radical is weak or limited by an activation free energy barrier.

The requirement of an anisotropic MFE sensitive to fractional variations in an Earth-strength (50 μT) magnetic field suggests the need for substantial chemical amplification of the ambient magnetic field. A radical bystander-based enhancement of the RPM might mitigate the problem of marginal anisotropic sensitivity,^55^ but it does not solve it. Rather, bystander-enhanced schemes remain tied to the essential recombination-based design of the RPM which employs the difference between separation and recombination reaction yields. Furthermore, although the introduction of a catalytic bystander radical might induce a larger MFE than the RPM alone, it does not rationalize the effects of weak magnetic fields on the biological production of reactive oxygen species.^18,81−86^ The accumulation of nuclear spin polarization realized via three-spin mixing, has also been suggested to deliver large MFEs in the presence of EED coupling.^87^ However, the necessity to retain nonequilibrium nuclear spin populations between subsequent photoexcitations questions the relevance of this proposal in vivo.^87^ A scavenger mechanism overcomes these issues, so a larger magnetosensitivity prevails as the primary-pair EED interaction may be compensated by that of a suitably placed scavenger radical. Scavenging also accommodates larger radical–radical distances (separating the primary pair) by obviating the need for significant recombination. From the RPM the mechanism inherits a comparable sensitivity to RF electromagnetic fields, as we demonstrated for a chosen system in the SI (Figure S12). These advantages may be offset by the extra reaction complexity introduced by radical scavenging, but the additional flexibility of this scenario nonetheless may generate sufficiently large MFEs to foster magnetoreception in vivo (in terms of ET rate constants and radical placements).

Although the additional complexity of a scavenger-mediated theory of magnetoreception could be considered detrimental, it is conceivable that natural selection may have provided the necessary structural and physiological optimizations to allow such a complex-but-efficient mechanism to emerge from existing design principles found in, for example, less-sensitive, RPM-based reactions evidenced in studies of the magnetosensitivity of cryptochromes in vitro. Our model predicts much larger MFEs than those of the RPM for immobilized radicals like cryptochrome’s. Significant scavenger-mediated MFEs of Γ ≫ 5% may arise naturally in cryptochrome in the geomagnetic field—while accounting for EED interactions even with the scavenger. Such a radical could be produced by reactions of reactive oxygen species (ROS) which are a well-known byproduct of cryptochrome reoxidation, or produced directly via photoreduction. Given that radical production has been linked to cryptochrome’s reactivity, it is plausible that evolutionary pressures have harnessed these secondary radical processes, exploiting scavenger-based MFEs for sensing and ultimately even navigation.

Here, we have predicted large maximum MFEs in a W_D_^•+^-scavenged model of *Cl*Cry4, for which the Trp-tetrad was proposed to be extended by an additional tyrosine (Tyr) residue implicated during photoreduction.^57^ In a recent experiment, it was found that site-directed mutagenesis of Tyr319 (Y_E_) to Asp319 resulted in a significant decrease in the of FAD-photoreduction quantum yield, attributed to the elimination of FAD-Tyr radical-pair formation, suggesting its relevance as extended electron transfer pathway. Conspicuously, we, here, identify an adjacent Tyr site (Tyr407, labeled Y_F_ in Figure 1) that may be well-suited to enable scavenger-based magnetosensitivity in *Cl*Cry4 by reducing W_D_^•+^. Such a process could, for example, be enabled by a coordinated, long-lived ascorbyl radical, previously generated in the photoreduction by via Y_E_. That this pathway of scavenger generation is in principle viable is also demonstrated in the work of Giovani et al. demonstrating long-lived tyrosine radicals (exceeding 100 ms in the absence of reductants) and their efficient reduction by external reductants on the time scale of milliseconds.^88^ Alternatively, the direct scavenging of the FAD-radical from these remote tyrosine sites could sustain sizable MFEs in the [FADH^•^/Z^•–^] models, as previously suggested, provided that the electronic coupling was very efficient, that is, β small.^64^ In view of the reductive cellular environment, this would require shielding of the tyrosine radicals from premature reduction, possibly through conformational changes or protein–protein interactions and association with appropriate signaling partners.

Although we shall not speculate further as to the radical scavenger and site identities, the prospect of a scavenger located somewhere near the terminus of the Trp-tetrad is particularly promising, as it could be generated in well-controlled manner, that is, the photoreduction process, and could furthermore return the spare electron via a long-distance ET-process, again possibly involving the Trp-tetrad. It is tempting to speculate that [FAD^•–^/W^•+^] and [FADH^•^/Z^•–^] model mechanisms could designate distinct steps in the same overall process. The oxidation products generated in the light-driven reduction process could then be used to generate stable radical scavengers poised to interact with a radical pair generated in the reoxidation process of FADH^–^ by molecular oxygen (forming FADH^•^ and superoxide). However, given that many chemical details are as yet unknown—not to mention the question of the structure of the protein when interacting with binding partners in vivo—the crucial issue at hand is not the actual site, but the principles needed to realize such exquisite sensitivities to weak magnetic fields in the presence of EED interactions. To this end, we emphasize that the radical pairs and scavenger molecules considered here are meant to inform and suggest future lines of inquiry while proposing likely candidate models for more detailed investigations, and these results should not yet be considered conclusive.

The tremendous MFEs > 70% predicted for the FAD-scavenged system support an idea that superoxide (e.g., formed in the oxidation cycle of FAD after photoreduction) could play a role in a chemical magnetoreceptive system. This highlights the question of the identity of the scavenger itself. We note that the scavenging reaction is known to be robust against Z^•–^-decoherence in the primary pair,^63^ thus providing a plausible basis for discussing the role of superoxide in the avian chemical compass despite its fast spin relaxation. Recognition that the rapid decoherence of a nearby Z_near_^•–^ can sometimes enhance MFEs lends credence to the notion that a fast-relaxing radical (such as O_2_^•–^) may be integrated in the reaction scheme consistently via a scavenging-mediated MFE.^64^ While large effects appear feasible, this also implicates the need for a novel three-radical reoxidation process, as a topic for future work.

In vivo, the indiscriminate production of free radicals as “signalling” (escape) products could influence homeostasis both via signaling molecules and through oxidative stress, indicating that radicals produced by a chemical compass mechanism need to be controlled, preferably traveling along designated ET pathways to be taken up by dedicated redox partners. Likewise, the magneto-sensor could be charged (by generating the fully reduced FADH^–^ and the scavenger radical) over time, poising it for discharge when triggered later. Such a scheme could address problems that are inherent to a stochastic quantum read-out, needed to infer the compass orientation, by allowing the swift accumulation of a large number of reaction events depending only on the MFE size. In contrast, the absence of electronic preloading could preclude use of a conventional RPM-based sensor during the night or other low-lighting conditions, discussed elsewhere.^89^ We overcome the faint-signal problem by introducing much larger-scale MFEs to allow for an accurate, low-latency sensor—poised for rapid chemical magnetoreception. In any case, it is critical that the outcome of the magnetosensitive chemical step should trigger the molecule for signaling. Therefore, establishing details of the sensor’s resting and activated states could be helpful to infer the characteristics of any subsequent chemical steps or structural rearrangements that are involved in sensing.

In summary, we have used extensive numerical simulations to illustrate the plausibility of a magnetochemical radical-scavenging mechanism under realistic physical conditions. This constitutes a fundamental step toward the resolution the previously unresolved question of how to sustain magnetic sensitivity in the presence of EED interactions that suppress MFEs in recombination-based sensors. Our predictions reveal that EED interactions need not be critically detrimental to MFEs in cryptochrome, and that the prospects for this radical biomagnetic sensor are not yet ruled out. To the contrary, we anticipate that our findings may inspire new efforts to uncover the true nature of the “cryptic” sensor underlying magnetochemical effects in biology by providing a previously unexplored line of investigation.

We suggest that tractable experiments could be built around the cyclic photoreduction and dark reoxidation of a cryptochrome in vitro. Weak magnetic fields could be applied selectively during the photoreduction or the dark reoxidation. The fluorescence during the photoreduction cycle could be used to readout the oxidation yield,^42^ or cavity-enhanced absorption^44^ could be used to detect the radical states. If three-radical processes are pertinent, as predicted, the MFEs will depend on the frequency of the photoreduction and reoxidation cycles, as any transient radicals will have finite lifetimes. This experiment could be carried out in the presence of radical scavengers, such as ascorbic acid, to periodically generate observable MFEs and their associated radical transients. Alternatively, the effects of stable free radicals on the MFEs could be probed. Experiments investigating this kind spin chemistry are being increasingly pursued.^90,91^ It will be necessary to ensure three-radical correlation rather than plainly exposing radical pairs to (multiple) scavenger radicals, which would abolish the MFEs.^92^ Thus, a certain degree of compartmentalization (e.g., in micelles), association (e.g., scavenger radical binding), or reduction of translational mobility will be required. These experiments would need to be accompanied by theoretical predictions of the general form realized here, being specifically adapted to the exact radical species under experimental consideration in cryptochrome or other biological macromolecules of interest for the practical application of chemical magnetoreception.

## Conclusions

Analysis of EED coupling in physically realistic RPM models revealed a “Catch-22” scenario: MFEs are suppressed by the intrusion of EED interactions if the primary radical pair is close enough together to allow adequate recombination, but become negligible if the primary pair is separated far enough apart to sufficiently reduce EED coupling (via the inverse-cube separation law of the EED interaction energy) due to the reduced recombination likelihood. An RPM mechanism might still provide a plausible scheme for low-field MFEs in the biological context (e.g., the isotropic MFEs of freely diffusing systems). However, its lack of resilience to EED interactions—facing the inefficiency of *J*/*D* cancellation schemes—casts doubt on its significance as the chemical basis for precise magnetic navigation in vivo.

Here, we have revisited predictions of anisotropic biochemical MFEs in the Earth’s magentic field, based on the RPM and its variants (such as the recently developed R3M^55^). These mechanisms seem unlikely to produce MFEs of more than a few percent, even under the best imaginable biochemical conditions, when physically realistic models that include EED interactions are considered. Although we have focused on the protein cryptochrome in our study, prototype radical reactions of the form considered herein may be encountered throughout a wide range of biological processes, which typically incorporate numerous hyperfine interactions, dipolar couplings between electrons, and potentially fast spin relaxation. Reactive oxygen species, frequently implicated in reactions of this kind, are particularly prone to rapid spin relaxation. Consequently, these characteristics cannot be summarily neglected, and must be considered (if not explicitly included) in studies of spin-biochemical reactions.

Although large magnetoreceptive effects in the protein cryptochrome are hypothesized to enable an exquisitely responsive geomagnetic compass sense in birds and other animals, we have demonstrated how it may not be practical to produce large, anisotropic MFEs in purely RPM-based mechanisms. On the other hand, radical models which include a third, *reactive* radical “scavenger” may in principle generate large MFEs, presenting the hope that they may be suitable for use in prototype chemical compass mechanisms—even in the presence of strong EED interactions, variable ET rates, and the prospect of substantial decoherence. Scavenger radicals could be independent molecules, cofactors bound to the cryptochrome substrate or other electroactive residues in cryptochrome, such as long-lived tyrosine radicals.

We have provided the first numerical evidence that the mechanism is robust in the presence of EED interactions, although favorable features of scavenged radical-pair systems were examined previously.^55,63,64^ Magnetic interactions between immobilized radicals are intrinsic to the electrons’ magnetic moments, and so cannot be readily omitted by design. Yet, these interactions have been neglected from a majority of studies so far, typically on account of the fair argument that such demanding calculations are not tractable for large radical spin systems. However, this simplification may have given too optimistic an assessment of the extent to which typical radical pair recombination reactions are sensitive to variations in the weak magnetic fields relevant to navigation and orientation by various organisms. We propose that these limitations may be overcome by a three-radical scavenging mechanism.

The model we propose is new insofar as we are unaware of any previous experimental evidence directly showing the magnetic influence of a radical scavenger on a biochemical MFE. In this context, these predictions warrant the further exploration of radical scavenger-based MFEs in biological, bioinspired, and synthetic molecules. Simulations indicate potential roles for sulfur-containing or aromatic residues located nearby the proposed primary radical pairs (and which not need exclude each other). In particular, we suggest a novel role for radical scavenger-enabled magnetic sensing, and suggest a possible scavenger site near the Cry C-terminal tail. We recommend the inclusion of all relevant physics, with explicit treatments of EED interactions and ET beyond the primary pair to investigate the possible involvement of a third radical. Future work may be aimed at developing experimentally testable predictions of conformational protein signaling states and identifying ET pathways most critical to cryptochrome signaling. Thus, scavenger-mediated models of cryptochrome-based magnetoreceptors are currently the only ones that are able to predict the substantial MFEs anticipated to enable the precision of the avian inclination compass. Models neglecting EED interactions may not be considered predictive of radical MFE-based sensors in vivo.

## Supporting Information Available

Details on the modeling approach, maximal magnetic field effects realized in models with unconstrained recombination rate constants, a comparison with R3M/RPM models, and additional data for scavenger-mediated MFEs, including a description of ideal scavenger locations (PDF)

## References

[ref1] Wiltschko R.; Wiltschko W. Magnetoreception. BioEssays 2006, 28, 157–168. 10.1002/bies.20363. 16435299

[ref2] Hore P. J.; Mouritsen H. The radical-pair mechanism of magnetoreception. Annu. Rev. Biophys. 2016, 45, 299–344. 10.1146/annurev-biophys-032116-094545. 27216936

[ref3] Johnsen S.; Lohmann K. J. Magnetoreception in animals. Phys. Today 2008, 61, 29–35. 10.1063/1.2897947.

[ref4] Nordmann G. C.; Hochstoeger T.; Keays D. A. Magnetoreception - A sense without a receptor. PLoS Biol. 2017, 15, e2003234 10.1371/journal.pbio.2003234. 29059181 PMC5695626

[ref5] Hiscock H. G.; Worster S.; Kattnig D. R.; Steers C.; Jin Y.; Manolopoulos D. E.; Mouritsen H.; Hore P. J. The quantum needle of the avian magnetic compass. Proc. Natl. Acad. Sci. U. S. A. 2016, 113, 4634–4639. 10.1073/pnas.1600341113. 27044102 PMC4855607

[ref6] Kominis I. K. The radical-pair mechanism as a paradigm for the emerging science of quantum biology. Mod. Phys. Lett. B 2015, 29, 1530013 10.1142/S0217984915300136.

[ref7] Kim Y.; Bertagna F.; D’Souza E. M.; Heyes D. J.; Johannissen L. O.; Nery E. T.; Pantelias A.; Sanchez-Pedreño Jimenez A.; Slocombe L.; Spencer M. G.; et al. Quantum biology: An update and perspective. Quantum Rep. 2021, 3, 80–126. 10.3390/quantum3010006.

[ref8] Kirschvink J. L.; Winklhofer M.; Walker M. M. Biophysics of magnetic orientation: Strengthening the interface between theory and experimental design. J. R. Soc., Interface 2010, 7, S179–S191. 10.1098/rsif.2009.0491.focus. 20071390 PMC2843999

[ref9] Nielsen C.; Kattnig D. R.; Sjulstok E.; Hore P. J.; Solov’yov I. A. Ascorbic acid may not be involved in cryptochrome-based magnetoreception. J. R. Soc., Interface 2017, 14, 20170657 10.1098/rsif.2017.0657. 29263128 PMC5746572

[ref10] Lee A. A.; Lau J. C.; Hogben H. J.; Biskup T.; Kattnig D. R.; Hore P. J. Alternative radical pairs for cryptochrome-based magnetoreception. J. R. Soc., Interface 2014, 11, 20131063 10.1098/rsif.2013.1063. 24671932 PMC4006233

[ref11] Ritz T.; Ahmad M.; Mouritsen H.; Wiltschko R.; Wiltschko W. Photoreceptor-based magnetoreception: optimal design of receptor molecules, cells, and neuronal processing. J. R. Soc., Interface 2010, 7, S135–S146. 10.1098/rsif.2009.0456.focus. 20129953 PMC2843994

[ref12] Cai J.; Guerreschi G. G.; Briegel H. J. Quantum control and entanglement in a chemical compass. Phys. Rev. Lett. 2010, 104, 220502 10.1103/PhysRevLett.104.220502. 20867156

[ref13] Hogben H. J.; Efimova O.; Wagner-Rundell N.; Timmel C. R.; Hore P. J. Possible involvement of superoxide and dioxygen with cryptochrome in avian magnetoreception: origin of Zeeman resonances observed by in vivo EPR spectroscopy. Chem. Phys. Lett. 2009, 480, 118–122. 10.1016/j.cplett.2009.08.051.

[ref14] Solov’yov I. A.; Schulten K. Magnetoreception through cryptochrome may involve superoxide. Biophys. J. 2009, 96, 4804–4813. 10.1016/j.bpj.2009.03.048. 19527640 PMC2712043

[ref15] Ritz T.; Adem S.; Schulten K. A model for photoreceptor-based magnetoreception in birds. Biophys. J. 2000, 78, 707–718. 10.1016/S0006-3495(00)76629-X. 10653784 PMC1300674

[ref16] Schulten K.; Swenberg C. E.; Weller A. A biomagnetic sensory mechanism based on magnetic field modulated coherent electron spin motion. Z. Phys. Chem. 1978, 111, 1–5. 10.1524/zpch.1978.111.1.001.

[ref17] Hammad M.; Albaqami M.; Pooam M.; Kernevez E.; Witczak J.; Ritz T.; Martino C.; Ahmad M. Cryptochrome mediated magnetic sensitivity in Arabidopsis occurs independently of light-induced electron transfer to the flavin. Photochem. Photobiol. Sci. 2020, 19, 341–352. 10.1039/C9PP00469F. 32065192

[ref18] Pooam M.; Arthaut L. D.; Burdick D.; Link J.; Martino C. F.; Ahmad M. Magnetic sensitivity mediated by the Arabidopsis blue-light receptor cryptochrome occurs during flavin reoxidation in the dark. Planta 2019, 249, 319–332. 10.1007/s00425-018-3002-y. 30194534

[ref19] Pakhomov A.; Bojarinova J.; Cherbunin R.; Chetverikova R.; Grigoryev P. S.; Kavokin K.; Kobylkov D.; Lubkovskaja R.; Chernetsov N. Very weak oscillating magnetic field disrupts the magnetic compass of songbird migrants. J. R. Soc., Interface 2017, 14, 20170364 10.1098/rsif.2017.0364. 28794163 PMC5582129

[ref20] Wiltschko R.; Ahmad M.; Nießner C.; Gehring D.; Wiltschko W. Light-dependent magnetoreception in birds: The crucial step occurs in the dark. J. R. Soc., Interface 2016, 13, 20151010 10.1098/rsif.2015.1010. 27146685 PMC4892254

[ref21] Nießner C.; Denzau S.; Stapput K.; Ahmad M.; Peichl L.; Wiltschko W.; Wiltschko R. Magnetoreception: Activated cryptochrome 1a concurs with magnetic orientation in birds. J. R. Soc., Interface 2013, 10, 20130638 10.1098/rsif.2013.0638. 23966619 PMC3785833

[ref22] Gegear R. J.; Foley L. E.; Casselman A.; Reppert S. M. Animal cryptochromes mediate magnetoreception by an unconventional photochemical mechanism. Nature 2010, 463, 804–808. 10.1038/nature08719. 20098414 PMC2820607

[ref23] Ritz T.; Wiltschko R.; Hore P. J.; Rodgers C. T.; Stapput K.; Thalau P.; Timmel C. R.; Wiltschko W. Magnetic compass of birds is based on a molecule with optimal directional sensitivity. Biophys. J. 2009, 96, 3451–3457. 10.1016/j.bpj.2008.11.072. 19383488 PMC2718301

[ref24] Steiner U. E.; Ulrich T. Magnetic field effects in chemical kinetics and related phenomena. Chem. Rev. 1989, 89, 51–147. 10.1021/cr00091a003.

[ref25] Kattnig D. R.; Nielsen C.; Solov’yov I. A. Molecular dynamics simulations disclose early stages of the photo-activation of cryptochrome 4. New J. Phys. 2018, 20, 083018 10.1088/1367-2630/aad70f.

[ref26] Wiltschko R.; Wiltschko W. Magnetoreception in birds. J. R. Soc., Interface 2019, 16, 20190295 10.1098/rsif.2019.0295. 31480921 PMC6769297

[ref27] Shaw J.; Boyd A.; House M.; Woodward R.; Mathes F.; Cowin G.; Saunders M.; Baer B. Magnetic particle-mediated magnetoreception. J. R. Soc., Interface 2015, 12, 20150499 10.1098/rsif.2015.0499. 26333810 PMC4614459

[ref28] Kavokin K.; Chernetsov N.; Pakhomov A.; Bojarinova J.; Kobylkov D.; Namozov B. Magnetic orientation of garden warblers (Sylvia borin) under 1.4 MHz radiofrequency magnetic field. J. R. Soc., Interface 2014, 11, 20140451 10.1098/rsif.2014.0451. 24942848 PMC4208380

[ref29] Wiltschko R.; Gehring D.; Denzau S.; Nießner C.; Wiltschko W. Magnetoreception in birds: II. Behavioural experiments concerning the cryptochrome cycle. J. Exp. Biol. 2014, 217, 4225–4228. 10.1242/jeb.110981. 25472973 PMC4254397

[ref30] Schwarze S.; Schneider N.-L.; Reichl T.; Dreyer D.; Lefeldt N.; Engels S.; Baker N.; Hore P. J.; Mouritsen H. Weak broadband electromagnetic fields are more disruptive to magnetic compass orientation in a night-migratory songbird (erithacus rubecula) than strong narrow-band fields. Front. Behav. Neurosci. 2016, 10.3389/fnbeh.2016.00055. PMC4801848 27047356

[ref31] Tomanova K.; Vacha M. The magnetic orientation of the antarctic amphipod Gondogeneia antarctica is cancelled by very weak radiofrequency fields. J. Exp. Biol. 2016, 219, 1717–1724. 10.1242/jeb.132878. 27026715

[ref32] Fedele G.; Green E. W.; Rosato E.; Kyriacou C. P. An electromagnetic field disrupts negative geotaxis in Drosophila via a CRY-dependent pathway. Nat. Commun. 2014, 5, 4391 10.1038/ncomms5391. 25019586 PMC4104433

[ref33] Albaqami M.; Hammad M.; Pooam M.; Procopio M.; Sameti M.; Ritz T.; Ahmad M.; Martino C. F. Arabidopsis cryptochrome is responsive to radiofrequency (rf) electromagnetic fields. Sci. Rep. 2020, 10, 11260 10.1038/s41598-020-67165-5. 32647192 PMC7347919

[ref34] Wang J.; Du X.; Pan W.; Wang X.; Wu W. Photoactivation of the cryptochrome/photolyase superfamily. J. Photochem. Photobiol., C 2015, 22, 84–102. 10.1016/j.jphotochemrev.2014.12.001.

[ref35] Bazalova O.; Kvicalova M.; Valkova T.; Slaby P.; Bartos P.; Netusil R.; Tomanova K.; Braeunig P.; Lee H.-J.; Sauman I.; et al. Cryptochrome 2 mediates directional magnetoreception in cockroaches. Proc. Natl. Acad. Sci. U. S. A. 2016, 113, 1660–1665. 10.1073/pnas.1518622113. 26811445 PMC4760799

[ref36] Dodson C. A.; Hore P. J.; Wallace M. I. A radical sense of direction: signalling and mechanism in cryptochrome magnetoreception. Trends Biochem. Sci. 2013, 38, 435–446. 10.1016/j.tibs.2013.07.002. 23938034

[ref37] Günther A.; Einwich A.; Sjulstok E.; Feederle R.; Bolte P.; Koch K.-W.; Solov’yov I. A.; Mouritsen H. Double-cone localization and seasonal expression pattern suggest a role in magnetoreception for European robin cryptochrome 4. Curr. Biol. 2018, 28, 211–223. 10.1016/j.cub.2017.12.003. 29307554

[ref38] Pinzon-Rodriguez A.; Bensch S.; Muheim R. Expression patterns of cryptochrome genes in avian retina suggest involvement of Cry4 in light-dependent magnetoreception. J. R. Soc., Interface 2018, 15, 20180058 10.1098/rsif.2018.0058. 29593090 PMC5908540

[ref39] Nohr D.; Franz S.; Rodriguez R.; Paulus B.; Essen L.-O.; Weber S.; Schleicher E. Extended electron-transfer in animal cryptochromes mediated by a tetrad of aromatic amino acids. Biophys. J. 2016, 111, 301–311. 10.1016/j.bpj.2016.06.009. 27463133 PMC4968396

[ref40] Müller P.; Yamamoto J.; Martin R.; Iwai S.; Brettel K. Discovery and functional analysis of a 4th electron-transferring tryptophan conserved exclusively in animal cryptochromes and (6–4) photolyases. Chem. Commun. 2015, 51, 15502–15505. 10.1039/C5CC06276D. 26355419

[ref41] Maeda K.; Robinson A. J.; Henbest K. B.; Hogben H. J.; Biskup T.; Ahmad M.; Schleicher E.; Weber S.; Timmel C. R.; Hore P. J. Magnetically sensitive light-induced reactions in cryptochrome are consistent with its proposed role as a magnetoreceptor. Proc. Natl. Acad. Sci. U. S. A. 2012, 109, 4774–4779. 10.1073/pnas.1118959109. 22421133 PMC3323948

[ref42] Kattnig D. R.; Evans E. W.; Déjean V.; Dodson C. A.; Wallace M. I.; Mackenzie S. R.; Timmel C. R.; Hore P. J. Chemical amplification of magnetic field effects relevant to avian magnetoreception. Nat. Chem. 2016, 8, 384–391. 10.1038/nchem.2447. 27001735

[ref43] Sheppard D. M. W.; Li J.; Henbest K. B.; Neil S. R. T.; Maeda K.; Storey J.; Schleicher E.; Biskup T.; Rodriguez R.; Weber S.; et al. Millitesla magnetic field effects on the photocycle of an animal cryptochrome. Sci. Rep. 2017, 7, 42228 10.1038/srep42228. 28176875 PMC5296725

[ref44] Xu J.; Jarocha L. E.; Zollitsch T.; Konowalczyk M.; Henbest K. B.; Richert S.; Golesworthy M. J.; Schmidt J.; Déjean V.; Sowood D. J. C.; et al. Magnetic sensitivity of cryptochrome 4 from a migratory songbird. Nature 2021, 594, 535–540. 10.1038/s41586-021-03618-9. 34163056

[ref45] Zwang T. J.; Tse E. C. M.; Zhong D.; Barton J. K. A compass at weak magnetic fields using thymine dimer repair. ACS Cent. Sci. 2018, 4, 405–412. 10.1021/acscentsci.8b00008. 29632887 PMC5879481

[ref46] Henbest K. B.; Maeda K.; Hore P. J.; Joshi M.; Bacher A.; Bittl R.; Weber S.; Timmel C. R.; Schleicher E. Magnetic-field effect on the photoactivation reaction of Escherichia coli DNA photolyase. Proc. Natl. Acad. Sci. U. S. A. 2008, 105, 14395–14399. 10.1073/pnas.0803620105. 18799743 PMC2567148

[ref47] Zeng Z.; Wei J.; Liu Y.; Zhang W.; Mabe T. Magnetoreception of photoactivated cryptochrome 1 in electrochemistry and electron transfer. ACS Omega 2018, 3, 4752–4759. 10.1021/acsomega.8b00645. 31458694 PMC6641772

[ref48] Bouly J. P.; Schleicher E.; Dionisio-Sese M.; Vandenbussche F.; Van Der Straeten D.; Bakrim N.; Meier S.; Batschauer A.; Galland P.; Bittl R.; et al. Cryptochrome blue light photoreceptors are activated through interconversion of flavin redox states. J. Biol. Chem. 2007, 282, 9383–9391. 10.1074/jbc.M609842200. 17237227

[ref49] Müller P.; Ahmad M. Light-activated cryptochrome reacts with molecular oxygen to form a flavin-superoxide radical pair consistent with magnetoreception. J. Biol. Chem. 2011, 286, 21033–21040. 10.1074/jbc.M111.228940. 21467031 PMC3122164

[ref50] Procopio M.; Ritz T. The reference-probe model for a robust and optimal radical-pair-based magnetic compass sensor. J. Chem. Phys. 2020, 152, 065104 10.1063/1.5128128. 32061231

[ref51] Player T. C.; Hore P. J. Viability of superoxide-containing radical pairs as magnetoreceptors. J. Chem. Phys. 2019, 151, 225101 10.1063/1.5129608. 31837685

[ref52] Atkins C.; Bajpai K.; Rumball J.; Kattnig D. R. On the optimal relative orientation of radicals in the cryptochrome magnetic compass. J. Chem. Phys. 2019, 151, 065103 10.1063/1.5115445.

[ref53] Hiscock H. G.; Kattnig D. R.; Manolopoulos D. E.; Hore P. J. Floquet theory of radical pairs in radiofrequency magnetic fields. J. Chem. Phys. 2016, 145, 124117 10.1063/1.4963793. 27782620

[ref54] O’Dea A. R.; Curtis A. F.; Green N. J. B.; Timmel C. R.; Hore P. J. Influence of dipolar interactions on radical pair recombination reactions subject to weak magnetic fields. J. Phys. Chem. A 2005, 109, 869–873. 10.1021/jp0456943. 16838958

[ref55] Babcock N. S.; Kattnig D. R. Electron-electron dipolar interaction poses a challenge to the radical pair mechanism of magnetoreception. J. Phys. Chem. Lett. 2020, 11, 2414–2421. 10.1021/acs.jpclett.0c00370. 32141754 PMC7145362

[ref56] Keens R. H.; Bedkihal S.; Kattnig D. R. Magnetosensitivity in dipolarly coupled three-spin systems. Phys. Rev. Lett. 2018, 121, 96001 10.1103/PhysRevLett.121.096001. 30230901

[ref57] Zoltowski B. D.; Chelliah Y.; Wickramaratne A.; Jarocha L.; Karki N.; Xu W.; Mouritsen H.; Hore P. J.; Hibbs R. E.; Green C. B.; et al. Chemical and structural analysis of a photoactive vertebrate cryptochrome from pigeon. Proc. Natl. Acad. Sci. U. S. A. 2019, 116, 19449 10.1073/pnas.1907875116. 31484780 PMC6765304

[ref58] Nohr D.; Paulus B.; Rodriguez R.; Okafuji A.; Bittl R.; Schleicher E.; Weber S. Determination of radical–radical distances in light-active proteins and their implication for biological magnetoreception. Angew. Chem., Int. Ed. 2017, 56, 8550–8554. 10.1002/anie.201700389. 28627073

[ref59] Hiscock H. G.; Mouritsen H.; Manolopoulos D. E.; Hore P. J. Disruption of magnetic compass orientation in migratory birds by radiofrequency electromagnetic fields. Biophys. J. 2017, 113, 1475–1484. 10.1016/j.bpj.2017.07.031. 28978441 PMC5627152

[ref60] Hong G.; Pachter R.; Essen L.-O.; Ritz T. Electron transfer and spin dynamics of the radical-pair in the cryptochrome from *Chlamydomonas reinhardtii* by computational analysis. J. Chem. Phys. 2020, 152, 065101 10.1063/1.5133019. 32061221

[ref61] Solov’yov I. A.; Chandler D. E.; Schulten K. Magnetic field effects in Arabidopsis thaliana cryptochrome-1. Biophys. J. 2007, 92, 2711–2726. 10.1529/biophysj.106.097139. 17259272 PMC1831705

[ref62] Efimova O.; Hore P. J. Role of exchange and dipolar interactions in the radical pair model of the avian magnetic compass. Biophys. J. 2008, 94, 1565–1574. 10.1529/biophysj.107.119362. 17981903 PMC2242753

[ref63] Kattnig D. R.; Hore P. J. The sensitivity of a radical pair compass magnetoreceptor can be significantly amplified by radical scavengers. Sci. Rep. 2017, 7, 11640 10.1038/s41598-017-09914-7. 28912470 PMC5599710

[ref64] Kattnig D. R. Radical-pair-based magnetoreception amplified by radical scavenging: resilience to spin relaxation. J. Phys. Chem. B 2017, 121, 10215–10227. 10.1021/acs.jpcb.7b07672. 29028342

[ref65] Letuta A. S.; Berdinskii V. L. Chemical Zeno effect–A new mechanism of spin catalysis in radical triads. Dokl. Phys. Chem. 2015, 463, 179–181. 10.1134/S0012501615080059.

[ref66] Kaulakys B.; Gontis V. Quantum anti-Zeno effect. Phys. Rev. A: At., Mol., Opt. Phys. 1997, 56, 1131 10.1103/PhysRevA.56.1131.

[ref67] Levy C.; Zoltowski B. D.; Jones A. R.; Vaidya A. T.; Top D.; Widom J.; Young M. W.; Scrutton N. S.; Crane B. R.; Leys D. Updated structure of Drosophila cryptochrome. Nature 2013, 495, E3–E4. 10.1038/nature11995. 23518567 PMC3694752

[ref68] Vaidya A. T.; Top D.; Manahan C. C.; Tokuda J. M.; Zhang S.; Pollack L.; Young M. W.; Crane B. R. Flavin reduction activates Drosophila cryptochrome. Proc. Natl. Acad. Sci. U. S. A. 2013, 110, 20455–20460. 10.1073/pnas.1313336110. 24297896 PMC3870761

[ref69] Gray H. B.; Winkler J. R. Long-range electron transfer. Proc. Natl. Acad. Sci. U. S. A. 2005, 102, 3534–3539. 10.1073/pnas.0408029102. 15738403 PMC553296

[ref70] Marcus R. A.; Sutin N. Electron transfers in chemistry and biology. Biochim. Biophys. Acta, Rev. Bioenerg. 1985, 811, 265–322. 10.1016/0304-4173(85)90014-X.

[ref71] Marcus R. A. Nonadiabatic processes involving quantum-like and classical-like coordinates with applications to nonadiabatic electron transfers. J. Chem. Phys. 1984, 81, 4494–4500. 10.1063/1.447418.

[ref72] Page C. C.; Moser C. C.; Chen X.; Dutton P. L. Natural engineering principles of electron tunnelling in biological oxidation-reduction. Nature 1999, 402, 47–52. 10.1038/46972. 10573417

[ref73] Moser C. C.; Anderson J. L. R.; Dutton P. L. Guidelines for tunneling in enzymes. Biochim. Biophys. Acta, Bioenerg. 2010, 1797, 1573–1586. 10.1016/j.bbabio.2010.04.441. PMC3509937 20460101

[ref74] Moser C. C.; Keske J. M.; Warncke K.; Farid R. S.; Dutton P. L. Nature of biological electron transfer. Nature 1992, 355, 796–802. 10.1038/355796a0. 1311417

[ref75] Kobylkov D.; Wynn J.; Winklhofer M.; Chetverikova R.; Xu J.; Hiscock H.; Hore P. J.; Mouritsen H. Electromagnetic 0.1–100 kHz noise does not disrupt orientation in a night-migrating songbird implying a spin coherence lifetime of less than 10 μs. J. R. Soc., Interface 2019, 16, 20190716 10.1098/rsif.2019.0716. 31847760 PMC6936046

[ref76] Langen R.; Colón J. L.; Casimiro D. R.; Karpishin T. B.; Winkler J. R.; Gray H. B. Electron tunneling in proteins: role of the intervening medium. JBIC, J. Biol. Inorg. Chem. 1996, 1, 221–225. 10.1007/s007750050046.

[ref77] Winkler J. R.; Gray H. B. Long-range electron tunneling. J. Am. Chem. Soc. 2014, 136, 2930–2939. 10.1021/ja500215j. 24499470 PMC3986022

[ref78] Lagunas A.; Guerra-Castellano A.; Nin-Hill A.; Díaz-Moreno I.; De la Rosa M. A.; Samitier J.; Rovira C.; Gorostiza P. Long distance electron transfer through the aqueous solution between redox partner proteins. Nat. Commun. 2018, 9, 5157 10.1038/s41467-018-07499-x. 30514833 PMC6279779

[ref79] Ratner M. A. Bridge-assisted electron transfer: effective electronic coupling. J. Phys. Chem. 1990, 94, 4877–4883. 10.1021/j100375a024.

[ref80] Kerpal C.; Richert S.; Storey J. G.; Pillai S.; Liddell P. A.; Gust D.; Mackenzie S. R.; Hore P. J.; Timmel C. R. Chemical compass behaviour at microtesla magnetic fields strengthens the radical pair hypothesis of avian magnetoreception. Nat. Commun. 2019, 10, 3707 10.1038/s41467-019-11655-2. 31420558 PMC6697675

[ref81] Van Huizen A. V.; Morton J. M.; Kinsey L. J.; Von Kannon D. G.; Saad M. A.; Birkholz T. R.; Czajka J. M.; Cyrus J.; Barnes F. S.; Beane W. S. Weak magnetic fields alter stem cell–mediated growth. Sci. Adv. 2019, 5, eaau7201 10.1126/sciadv.aau7201. 30729158 PMC6353618

[ref82] Lai H. Exposure to static and extremely-low frequency electromagnetic fields and cellular free radicals. Electromagn. Biol. Med. 2019, 38, 231–248. 10.1080/15368378.2019.1656645. 31450976

[ref83] Sherrard R. M.; Morellini N.; Jourdan N.; El-Esawi M.; Arthaut L.-D.; Niessner C.; Rouyer F.; Klarsfeld A.; Doulazmi M.; Witczak J.; et al. Low-intensity electromagnetic fields induce human cryptochrome to modulate intracellular reactive oxygen species. PLoS Biol. 2018, 16, e2006229 10.1371/journal.pbio.2006229. 30278045 PMC6168118

[ref84] Wang H.; Zhang X. Magnetic fields and reactive oxygen species. Int. J. Mol. Sci. 2017, 18, 2175 10.3390/ijms18102175. PMC5666856 29057846

[ref85] Novikov V. V.; Yablokova E. V.; Fesenko E. E. The effect of weak magnetic fields on the production of reactive oxygen species in neutrophils. Biophysics 2016, 61, 959–962. 10.1134/S0006350916060208.

[ref86] Usselman R. J.; Hill I.; Singel D. J.; Martino C. F. Spin biochemistry modulates reactive oxygen species (ROS) production by radio frequency magnetic fields. PLoS One 2014, 9, e93065 10.1371/journal.pone.0093065. 24681944 PMC3969378

[ref87] Wong S. Y.; Solov’yov I. A.; Hore P. J.; Kattnig D. R. Nuclear polarization effects in cryptochrome-based magnetoreception. J. Chem. Phys. 2021, 154, 035102 10.1063/5.0038947. 33499614

[ref88] Giovani B.; Byrdin M.; Ahmad M.; Brettel K. Light-induced electron transfer in a cryptochrome blue-light photoreceptor. Nat. Struct. Mol. Biol. 2003, 10, 489–490. 10.1038/nsb933. 12730688

[ref89] Hiscock H. G.; Hiscock T. W.; Kattnig D. R.; Scrivener T.; Lewis A. M.; Manolopoulos D. E.; Hore P. J. Navigating at night: fundamental limits on the sensitivity of radical pair magnetoreception under dim light. Q. Rev. Biophys. 2019, 52, e9 10.1017/S0033583519000076. 31637984

[ref90] Rugg B. K.; Krzyaniak M. D.; Phelan B. T.; Ratner M. A.; Young R. M.; Wasielewski M. R. Photodriven quantum teleportation of an electron spin state in a covalent donor–acceptor–radical system. Nat. Chem. 2019, 11, 981–986. 10.1038/s41557-019-0332-8. 31548665

[ref91] Bagryansky V. A.; Borovkov V. I.; Bessmertnykh A. O.; Tretyakova I. S.; Beregovaya I. V.; Molin Y. N. Interaction of spin-correlated radical pair with a third radical: Combined effect of spin-exchange interaction and spin-selective reaction. J. Chem. Phys. 2019, 151, 224308 10.1063/1.5127812. 31837666

[ref92] Borovkov V. I.; Ivanishko I. S.; Bagryansky V. A.; Molin Y. N. Spin-selective reaction with a third radical destroys spin correlation in the surviving radical pairs. J. Phys. Chem. A 2013, 117, 1692–1696. 10.1021/jp312253v. 23421480

[ref93] Pettersen E. F.; Goddard T. D.; Huang C. C.; Couch G. S.; Greenblatt D. M.; Meng E. C.; Ferrin T. E. UCSF Chimera–a visualization system for exploratory research and analysis. J. Comput. Chem. 2004, 25, 1605–1612. 10.1002/jcc.20084. 15264254

