# Supplementary material for: Radical Scavenging Could Answer the Challenge Posed by Electron–Electron Dipolar Interactions in the Cryptochrome Compass Model
Source: JACS Au. 2021 Oct 5;1(11):2033–46. doi: 10.1021/jacsau.1c00332 (PMC8611662; doi:10.1021/jacsau.1c00332)
Supplement: Supplementary file 1 — au1c00332_si_001.pdf [file au1c00332_si_001.pdf]

Supporting Information: Radical Scavenging Could Answer the Challenge Posed by Electron-Electron Dipolar Interactions in the Cryptochrome Compass Model

Nathan Sean Babcock[‡,†] and Daniel R. Kattnig[*,‡]

† *Quantum Biology Laboratory, Howard University, 2400 Sixth Street NW, Washington, District of Columbia, 20059, United States of America*

‡ *Living Systems Institute and Department of Physics University of Exeter, Stocker Road, Exeter, EX4 4QD, United Kingdom*

E-mail: *d.r.kattnig@exeter.ac.uk

Phone: +44 (0) 1392 72 7479

Spin dynamics simulations

Dynamics of the spin density $\hat{\rho}(t)$ are described by the Liouville-von Neumann equation,

$$\frac{d}{dt}\hat{\rho}(t) = -\frac{\mathrm{i}}{\hbar}\left[\hat{H}, \hat{\rho}(t)\right] - k_\mathrm{f}\,\hat{\rho}(t) - \frac{k_\mathrm{X}}{2}\left\{\hat{P}_\mathrm{S}^{ab}, \hat{\rho}(t)\right\}\,, \tag{S1}$$

where the time-independent Hamiltonian may be written as sum of energy operators:

$$\hat{H} = \hat{H}_\mathrm{Zee} + \hat{H}_\mathrm{dip} + \hat{H}_\mathrm{hf} + \hat{H}_\mathrm{ex}\,, \tag{S2}$$

including magnetic Zeeman effects $\hat{H}_\mathrm{Zee}$, electron-electron dipolar (EED) interactions $\hat{H}_\mathrm{dip}$, electron-nuclear "hyperfine" coupling $\hat{H}_\mathrm{hf}$, and electron-electron exchange $\hat{H}_\mathrm{ex}$. We neglect decoherence processes, assumed slow relative to the lifetime of the radical pair, as reflected in the rate constants for scavenging k_X and escape k_f. As usual, square brackets $[\,,]$ denote commutation, whereas brace brackets $\{\,,\}$ indicate anti-commutation of the enclosed terms.

In detail, the Zeeman Hamiltonian $\hat{H}_\mathrm{Zee} = \sum_i g_i\,\mu_\mathrm{B}\,\hat{\vec{S}}_i \cdot \vec{B}$ describes interaction of the magnetic field denoted $\vec{B}$ with each radical i's spin angular momentum (expressed as unitless vector operators $\hat{\vec{S}}_i$), where μ_B is the Bohr magneton. The dipolar Hamiltonian $\hat{H}_\mathrm{dip}$ defines the energy of the EED interactions,

$$\hat{H}_\mathrm{dip} = -\sum_{i>j}^{m} D_{ij}(r_{ij})\left(3(\hat{\vec{S}}_i \cdot \vec{u}_{ij})(\hat{\vec{S}}_j \cdot \vec{u}_{ij}) - (\hat{\vec{S}}_i \cdot \hat{\vec{S}}_j)\right) \tag{S3}$$

where $D_{ij}(r_{ij}) = \mu_0\,g_i\,g_j\,\mu_\mathrm{B}^2/(4\pi|\vec{r}_{ij}|^3)$, $\vec{r}_{ij} = \vec{r}_j - \vec{r}_i$ gives the displacement from radical i to j where $\vec{u}_{ij} = \vec{r}_{ij}/|\vec{r}_{ij}|$ is the corresponding unit vector, g_i and g_j designate electronic g-factors, and μ_0 denotes the magnetic permeability of free space. The hyperfine Hamiltonian $\hat{H}_\mathrm{hf}$ defines the energy of interaction of the ith electron spin with the n_i magnetic nuclei (enumerated as ℓ_i) within the radical i:

$$\hat{H}_\mathrm{hf} = \sum_i \hat{\vec{S}}_i \cdot \left(\sum_{\ell_i=1}^{n_i} \mathbb{A}_{i\ell} \cdot \hat{\vec{I}}_{\ell_i}\right). \tag{S4}$$

The anisotropic hyperfine coupling tensors $\mathbb{A}_{i\ell}$ define the electron-nuclear magnetic couplings where they are present. The exchange Hamiltonian is of the form $\hat{H}_{\mathrm{ex}} = \sum_{i>j} J_{ij} \left(2\hat{\vec{S}}_i \cdot \hat{\vec{S}}_j + \frac{1}{2}\right)$. The projector $\hat{P}_{\mathrm{S}}^{ab}$ defines the singlet state of any two radicals a and b. Recombination is recovered in the R3M case where $(a, b) = (1, 2)$, whereas scavenging of either of either member of the radical pair is modeled otherwise, *i.e.*, either $(a, b) = (1, 3)$ or $(a, b) = (2, 3)$.

We modeled hyperfine (HF) coupling interactions up to a total of four magnetic nuclei. For the [$\mathrm{FAD}^{\bullet-}$ / $\mathrm{W}^{\bullet+}$] simulations, two hyperfine interactions were retained each for the FAD and W radical sites. For *Dm*Cry, we have used the following previously-calculated hyperfine tensors:[1]

$$\mathbb{A}_{\mathrm{FAD\ N_5}} = \begin{bmatrix} -0.099 & 0.003 & 0 \\ 0.003 & -0.087 & 0 \\ 0. & 0. & 1.757 \end{bmatrix} \mathrm{mT},$$

$$\mathbb{A}_{\mathrm{FAD\ N_{10}}} = \begin{bmatrix} -0.015 & 0.002 & 0 \\ 0.002 & -0.024 & 0 \\ 0 & 0 & 0.605 \end{bmatrix} \mathrm{mT},$$

$$\mathbb{A}_{\mathrm{W_C\ N_1}} = \begin{bmatrix} -0.034 & 0.092 & -0.135 \\ 0.092 & 0.330 & -0.532 \\ -0.135 & -0.532 & 0.668 \end{bmatrix} \mathrm{mT},$$

$$\mathbb{A}_{\mathrm{W_C\ H_1}} = \begin{bmatrix} -0.992 & -0.209 & -0.200 \\ -0.209 & -0.263 & 0.280 \\ -0.200 & 0.280 & -0.540 \end{bmatrix} \mathrm{mT},$$

$$\mathbb{A}_{\mathrm{W_D\ N_1}} = \begin{bmatrix} -0.048 & 0.132 & 0.013 \\ 0.132 & 1.056 & 0.103 \\ 0.0134 & 0.103 & -0.043 \end{bmatrix} \mathrm{mT},$$

$$\mathbb{A}_{\mathrm{W_D\ H_1}} = \begin{bmatrix} -0.678 & -0.050 & 0.516 \\ -0.050 & -0.693 & -0.081 \\ 0.516 & -0.081 & -0.425 \end{bmatrix} \mathrm{mT}.$$

For *Cl*Cry4, the flavin hyperfine tensors were as given above, whereas the tryptophan tensors were rotated to reflect its altered relative orientation in this protein:

$$\mathbb{A}_{\mathrm{W_C\ N_1}} = \begin{bmatrix} -0.053 & -0.059 & 0.046 \\ -0.059 & 0.564 & -0.565 \\ 0.046 & -0.565 & 0.453 \end{bmatrix} \mathrm{mT},$$

$$\mathbb{A}_{\mathrm{W_C\ H_1}} = \begin{bmatrix} -1.000 & -0.206 & -0.193 \\ -0.206 & -0.442 & 0.307 \\ -0.193 & 0.307 & -0.352 \end{bmatrix} \mathrm{mT},$$

$$\mathbb{A}_{\mathrm{W_D\ N_1}} = \begin{bmatrix} -0.063 & -0.0141 & -0.001 \\ -0.014 & 1.064 & 0.138 \\ -0.001 & 0.138 & -0.036 \end{bmatrix} \mathrm{mT},$$

$$\mathbb{A}_{\mathrm{W_D\ H_1}} = \begin{bmatrix} -0.697 & -0.062 & 0.512 \\ -0.062 & -0.704 & -0.026 \\ 0.512 & -0.026 & -0.394 \end{bmatrix} \mathrm{mT}.$$

For the flavin semiquinone, the following three tensors were used:

$$\mathbb{A}_{\mathrm{FAD\ N_5}} = \begin{bmatrix} -2.431 & -0.068 & -0.363 \\ -0.068 & -2.445 & 0.306 \\ -0.363 & 0.306 & 43.53 \end{bmatrix} \mathrm{MHz},$$

$$\mathbb{A}_{\mathrm{FAD\ N_{10}}} = \begin{bmatrix} 0.494 & 0.068 & 2.101 \\ 0.068 & -0.016 & -0.209 \\ 2.101 & -0.209 & 23.10 \end{bmatrix} \mathrm{MHz},$$

$$\mathbb{A}_{\mathrm{FAD\ H_5}} = \begin{bmatrix} -2.380 & 1.872 & 0.167 \\ 1.872 & -40.64 & 0.081 \\ 0.167 & 0.081 & -27.87 \end{bmatrix} \mathrm{MHz}.$$

The position of the scavenger radical was varied in three dimensions by assuming a virtual "shell" of possible locations on the vertices of a sphere-like geodesic polyhedron of radius R, while varying R. For each type of scavenger reaction (scavenging of the anion or cation of the primary radical pair), the polyhedron was centred around the scavenger's target radical. We initially sampled scavenger coordinates at $\Delta R = 1\,\text{Å}$ radial increments, recursively adjusting the radius increment and number of vertices to generate a smooth

MFE function Γ for each tunneling decay constant β tested. The "R3M"[1] recombination-based systems were approximated by simulating primary-pair recombination with a third, unreactive "bystander" radical nearby.

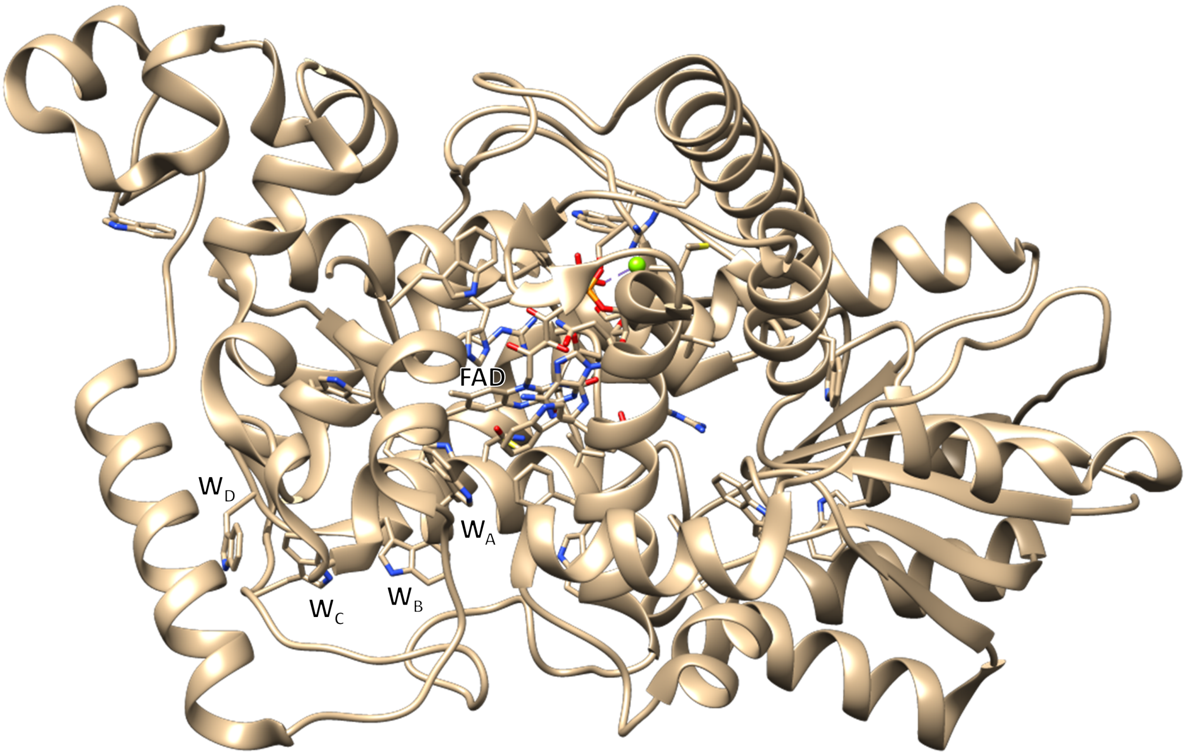

Figure S1: Graphical representation of *Dm*Cry4 (PDB ID: 4GU5)[2] as a ribbon diagram.[3] Labels indicate the FAD prosthetic group and Trp terad residues, superimposed on the protein ribbon to illustrate their positions in the molecule.

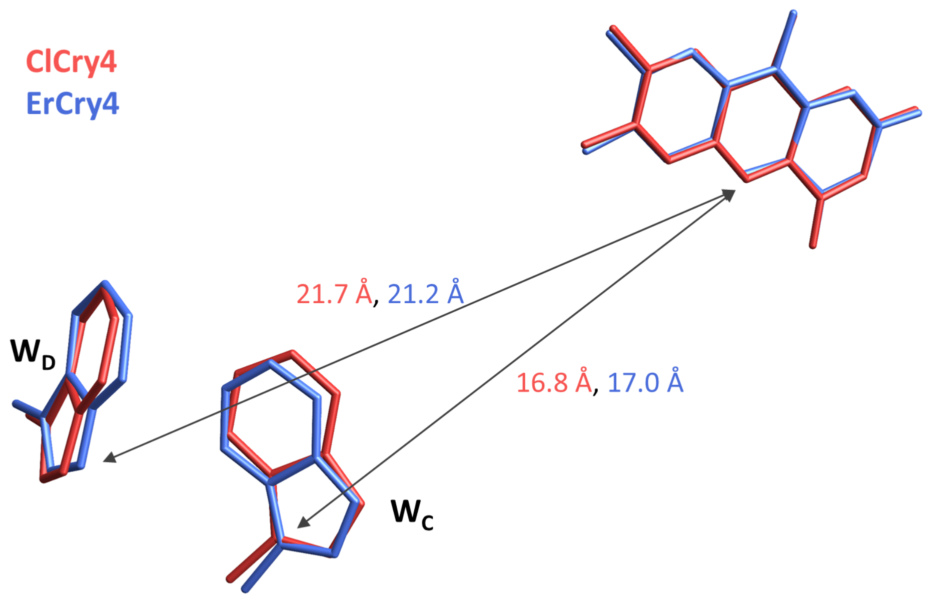

Figure S2: Pertinent residues in the crystal structure of *Cl*Cry4 (red) and the homology model of *Er*Cry4 from Ref. [4] (blue). The root-mean-square displacement (RMSD) of the relevant part of both of these structures amounts to only 1.2 Å and the differences in inter-radical distances are negligible (compared to their fluctuations as assessed in molecular dynamics simulation). Note in particular that *Dm*Cry and *Cl*Cry4 too differ by a RMSD of 1.2 Å, while the simulated MFEs of these systems are practically identical (*cf.* Fig. 2 and S8). No intrinsic enhancement of magnetosensitvity is expected for *Er*Cry4 within the constraints of the suggested model. The superiority recently established for *Er*Cry4 *in vitro* appears to be the result of tuned rate constants.[5] However, the rate constants established there do not permit substantial magnetosensitivity in the geomagnetic field. Changes in the relative radical orientation are likewise not expected to dramatically boost the magnetosensitivity.[6]

Table S1: Summary of three-radical reactions. The table shows the initial spin configuration of the *geminate* radical pair, the charge recombination mechanism, and the reaction scheme as given in the main manuscript. Bystander-enhanced schemes (in the top two rows) recover the RPM in the large-distance limit of the distance from the primary pair to bystander $B^\bullet$.

Radical Trio Notation	*Geminate* Pair	Mechanism	Scheme
($FAD^{\bullet-}$ / $W^{\bullet+}$) / $B^\bullet$	Singlet	Recombination	2
($FADH^\bullet$ / $Z^{\bullet-}$) / $B^\bullet$	Triplet	Recombination	2
$FAD^{\bullet-}$ / ($W^{\bullet+}$ / $S^\bullet$)	Singlet	Scavenging	3
($S^\bullet$ / $FAD^{\bullet-}$) / $W^{\bullet+}$	Singlet	Scavenging	3
$FADH^\bullet$ / ($Z^{\bullet-}$ / $S^\bullet$)	Triplet	Scavenging	3
($S^\bullet$ / $FADH^\bullet$) / $Z^{\bullet-}$	Triplet	Scavenging	3

Unconstrained models

We began by carrying out a systematic exploration of the reaction phase space, setting $k_{12} = 0$ and independently varying k_{13} and k_{23} for a scavenger radical positioned at one of a few choice locations around the surface of *Dm*Cry, but preliminary results did not suggest a likelihood of finding a single, joint optimum MFE for some specific values of $\{k_{13}, k_{23}\}$. So, as an alternative approach, we next set $k_{12} = k_{23} = 0$ for the ($S^{\bullet}$ / $FAD^{\bullet -}$) / $W^{\bullet +}$ reaction in *Dm*Cry, systematically considering a wide range of possible values for k_{13} while exploring possible scavenger positions over the whole volume of space within 200 Å of the FAD cofactor. To our surprise, a global maximum MFE anisotropy Γ_{max} was obtained using the value $k_{13} \approx 0.03\,\text{ns}^{-1}$ in the asymptotic limit as $R \to \infty$.

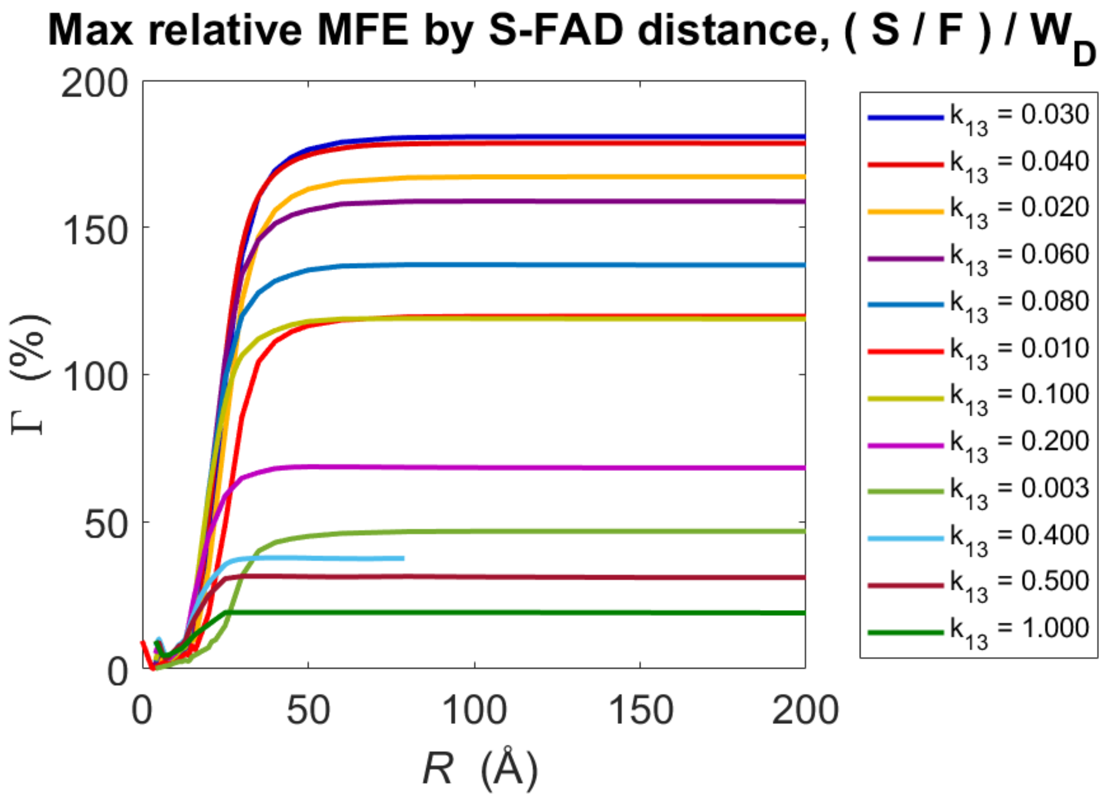

Figure S3: This plot shows the maximum anisotropic magnetic field effect Γ as a function the distance from the scavenger to its target FAD in the molecule *Dm*Cry.

Table S2: Unconstrained optimal relative MFEs $\Gamma^{\text{opt}}_{\text{max}}$, with corresponding scavenger-radical distances $R^{\text{opt}}_{\text{max}} \leq 100\text{Å}$ and rates $k^{\text{opt}}_{\text{X}}$. For the $\text{FAD}^{\bullet-}$ / $\text{W}^{\bullet+}$ radical pairs, we have studied the relative orientations as found in *Cl*Cry4 and *Dm*Cry. The $\text{FADH}^{\bullet}$ / $\text{Z}^{\bullet-}$ models are applicable to both species. ET rates are given in GHz. Brackets indicate which radical is being scavenged by $\text{S}^{\bullet}$ (*cf* Table S1). For comparison, see also Figures S2 - S5.

Radical Trio	*Dm*Cry $\Gamma^{\text{opt}}_{\text{max}}$	*Dm*Cry $R^{\text{opt}}_{\text{max}}$	*Dm*Cry k_{X}	*Cl*Cry4 $\Gamma^{\text{opt}}_{\text{max}}$	*Cl*Cry4 $R^{\text{opt}}_{\text{max}}$	*Cl*Cry4 k_{X}
$\text{FAD}^{\bullet-}$ / ($\text{W}^{\bullet+}_{\text{C}}$ / $\text{S}^{\bullet}$)	45%	70Å	0.03	44%	75Å	0.03
$\text{FAD}^{\bullet-}$ / ($\text{W}^{\bullet+}_{\text{D}}$ / $\text{S}^{\bullet}$)	53%	80Å	0.03	52%	90Å	0.03
$\text{FADH}^{\bullet}$ / ($\text{Z}^{\bullet-}_{\text{near}}$ / $\text{S}^{\bullet}$)	107%	100Å	0.03	107%	100Å	0.03
$\text{FADH}^{\bullet}$ / ($\text{Z}^{\bullet-}_{\text{far}}$ / $\text{S}^{\bullet}$)	147%	45Å	0.03	147%	45Å	0.03
($\text{S}^{\bullet}$ / $\text{FAD}^{\bullet-}$) / $\text{W}^{\bullet+}_{\text{C}}$	162%	100Å	0.03	158%	100Å	0.03
($\text{S}^{\bullet}$ / $\text{FAD}^{\bullet-}$) / $\text{W}^{\bullet+}_{\text{D}}$	181%	100Å	0.03	179%	100Å	0.03
($\text{S}^{\bullet}$ / $\text{FADH}^{\bullet}$) / $\text{Z}^{\bullet-}_{\text{near}}$	104%	100Å	0.03	104%	100Å	0.03
($\text{S}^{\bullet}$ / $\text{FADH}^{\bullet}$) / $\text{Z}^{\bullet-}_{\text{far}}$	89%	100Å	0.03	89%	100Å	0.03

Subsequently, we developed a rough three-parameter approximation of the optimal k_{13}, k^{opt}_{13}, intended to optimize the resulting Γ over all space for the reaction ($\text{S}^{\bullet}$ / $\text{FAD}^{\bullet-}$) / $\text{W}^{\bullet+}$ in the *Dm*Cry system:

$$k^{\text{opt}}_{13} \approx [\,0.03 + 6 \cdot \exp(-2 \cdot R/9)\,]\ \text{ns}^{-1}\,. \tag{S5}$$

Although the expression for k^{opt}_{13} given in eq. (S5) cannot be characterized as an exactly optimized function for all Rs, we found that it nevertheless provided a sense of asymptotically optimal behavior in the MFE, while delivering large MFEs at modest values of R.

When eq. (S5) was applied as the ET rate constant in the other scavenger-reaction systems listed in Table S2, it uniformly predicted large "optimal" values of the resulting MFEs in the asymptotic limit $R \to \infty$, where substantial scavenger-mediated ET rates could not be considered plausible indicators of MFE-mediating scavenging rates in realistic biological systems. *Ad hoc* preliminary findings obtained from applying eq. (S5) in the other model systems indicated that unconstrained optimizations of the scavenging rate in the asymptotic distance limit would not lead to realistic MFE predictions—a hypothesis which

was later borne out in the results of the studies we performed as a consequence using ET rates bounded by Marcus theory (*viz.* Figs. S3, S4, S5).

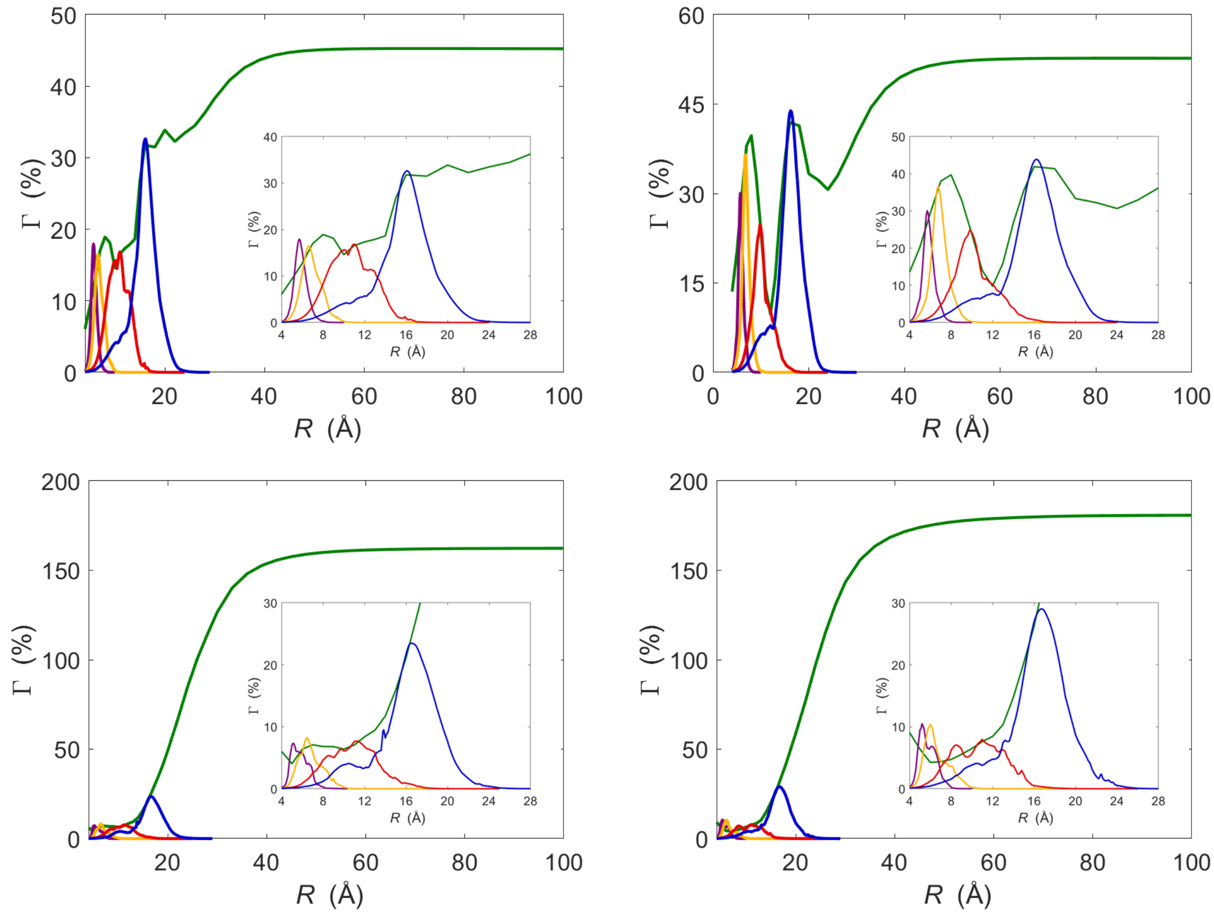

Figure S4: Top figures show $FAD^{\bullet-}$ / ($W_C^{\bullet+}$ / $S^{\bullet}$) [left] and $FAD^{\bullet-}$ / ($W_D^{\bullet+}$ / $S^{\bullet}$) [right]. Bottom figures show ($S^{\bullet}$ / $FAD^{\bullet-}$) / $W_C^{\bullet+}$ [left] and ($S^{\bullet}$ / $FAD^{\bullet-}$) / $W_D^{\bullet+}$ [right].

Plots show the maximal Γ as a function of the inter-radical distance R obtained for *Dm*Cry using eq. (S5) (green), superimposed on the maximal Γ realizable for activationless ET for four tunneling media: covalently-bound (blue), typical protein (red), "soft" vacuum (yellow), and "hard" vacuum (purple), shown up close in each inset plot.

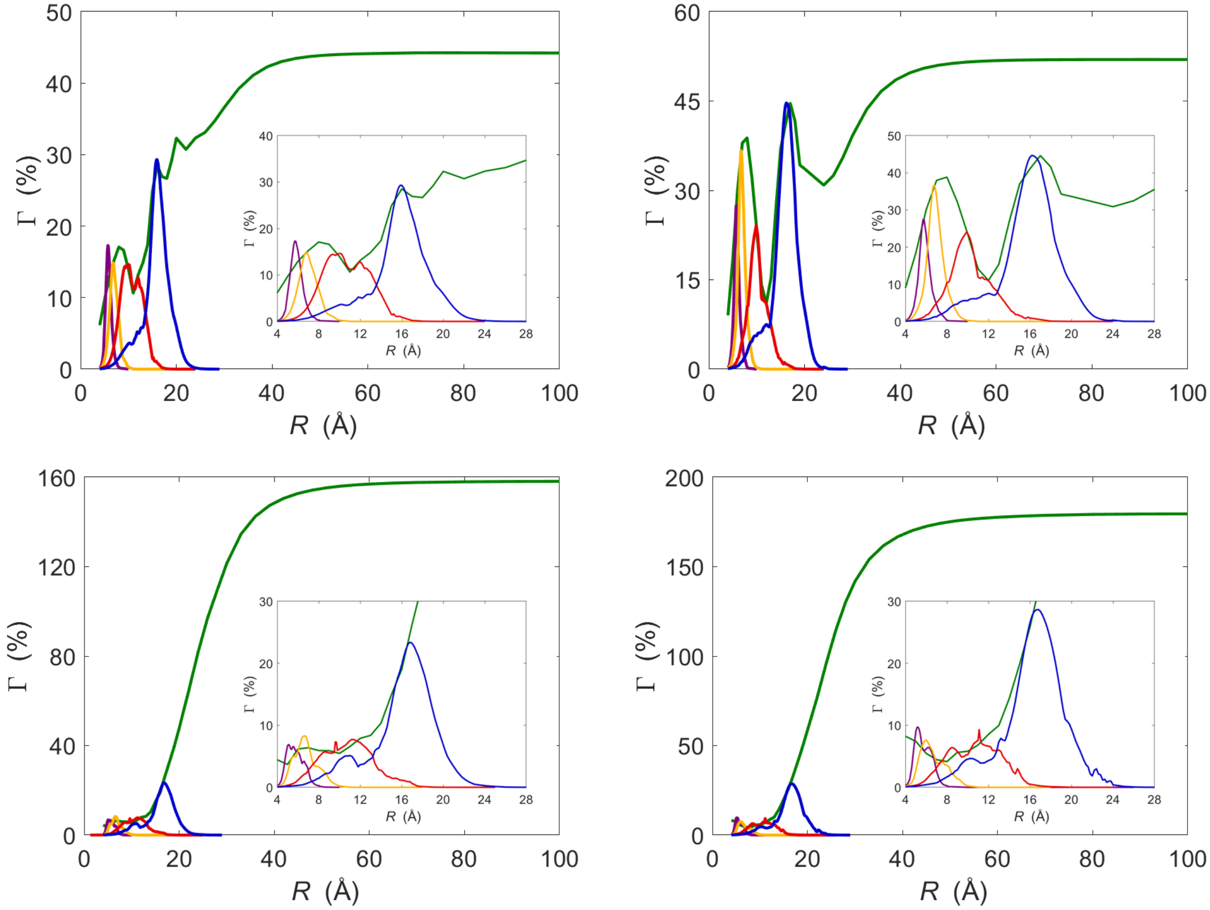

Figure S5: Top figures show $\mathrm{FAD}^{\bullet-}$ / ($\mathrm{W}_{\mathrm{C}}^{\bullet+}$ / $\mathrm{S}^{\bullet}$) [left] and $\mathrm{FAD}^{\bullet-}$ / ($\mathrm{W}_{\mathrm{D}}^{\bullet+}$ / $\mathrm{S}^{\bullet}$) [right]. Bottom figures show ($\mathrm{S}^{\bullet}$ / $\mathrm{FAD}^{\bullet-}$) / $\mathrm{W}_{\mathrm{C}}^{\bullet+}$ [left] and ($\mathrm{S}^{\bullet}$ / $\mathrm{FAD}^{\bullet-}$) / $\mathrm{W}_{\mathrm{D}}^{\bullet+}$ [right].

Plots show estimates of Γ predicted for *Cl*Cry4 using eq. (S5) (green), laid over the values fit for the various parameters based on activationless ET for four tunneling media: covalently-bound (blue), typical protein (red), “soft” vacuum (yellow), and “hard” vacuum (purple), shown up close in each inset plot.

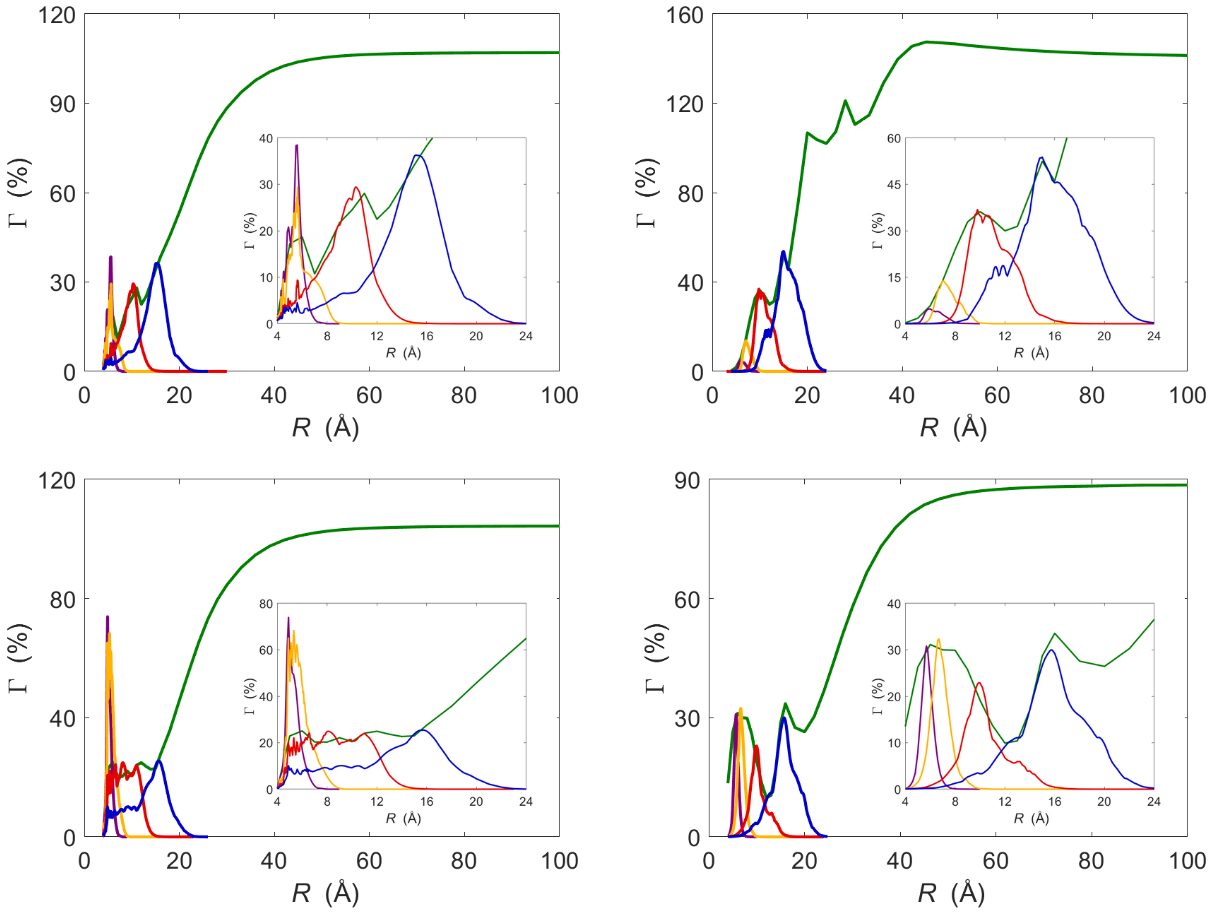

Figure S6: Top figures show FADH$^\bullet$ / ($Z^{\bullet-}_{near}$ / $S^\bullet$) [left] and FADH$^\bullet$ / ($Z^{\bullet-}_{far}$ / $S^\bullet$) [right]. Bottom figures show ($S^\bullet$ / FADH$^\bullet$) / $Z^{\bullet-}_{far}$ [left] and ($S^\bullet$ / FADH$^\bullet$) / $Z^{\bullet-}_{far}$ [right].

Plots show estimates of Γ obtained by using eq. (S5) (green), laid over the values fit for the various parameters based on activationless ET for four tunneling media: covalently-bound (blue), typical protein (red), "soft" vacuum (yellow), and "hard" vacuum (purple), shown up close in each inset plot.

Bystander-mediated Relative MFEs (R3M) and the RPM

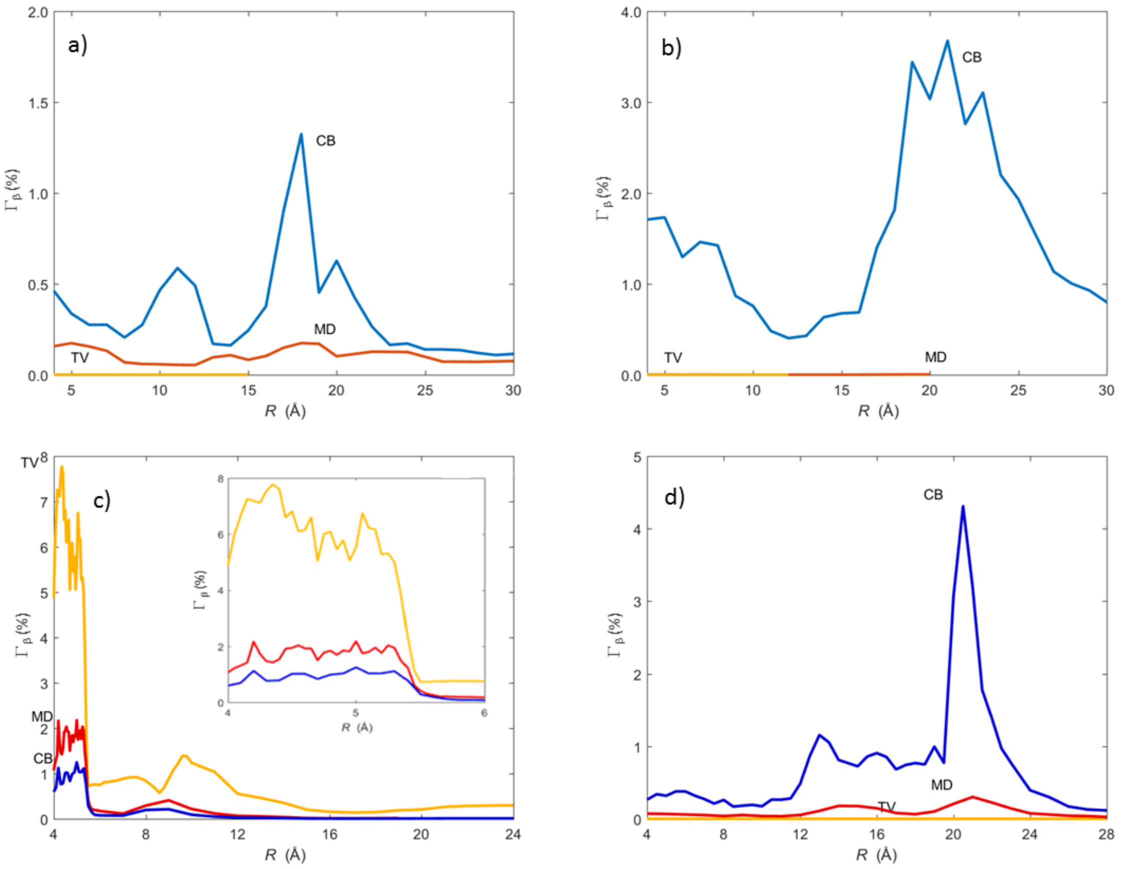

Figure S7: In order to compare the original RPM and its derivative the R3M to the scavenging-mediated mechanism on equal footing, we carried out an additional set of simulations of recombination-based MFEs in cryptochrome employing ET constraints like those used to bound the model of scavenger-mediated MFEs. These are based on activationless ET through three tunneling media: covalently-linked (CB, $\beta = 0.9\text{Å}^{-1}$) shown in blue, typical protein (MD, $\beta = 1.4\text{Å}^{-1}$) in red, and "soft" vacuum (TV, $\beta = 2.8\text{Å}^{-1}$) in yellow. The RPM model proper is recovered from the R3M as the bystander is placed sufficiently far away from the spin-polarized radical-pair to render the bystander's influence negligible, where the resulting MFE generally becomes negligible as well. The panels give the maximal relative anisotropy as a function of the bystander-flavin distance for a) ($\text{FAD}^{\bullet-}$ / $\text{W}_\text{C}^{\bullet+}$) / $\text{B}^\bullet$; b) ($\text{FAD}^{\bullet-}$ / $\text{W}_\text{D}^{\bullet+}$) / $\text{B}^\bullet$; c) ($\text{FADH}^\bullet$ / $\text{Z}_\text{near}^{\bullet-}$) / $\text{B}^\bullet$; and d) ($\text{FADH}^\bullet$ / $\text{Z}_\text{far}^{\bullet-}$) / $\text{B}^\bullet$.

Scavenger-mediated Relative MFEs in *Dm*Cry

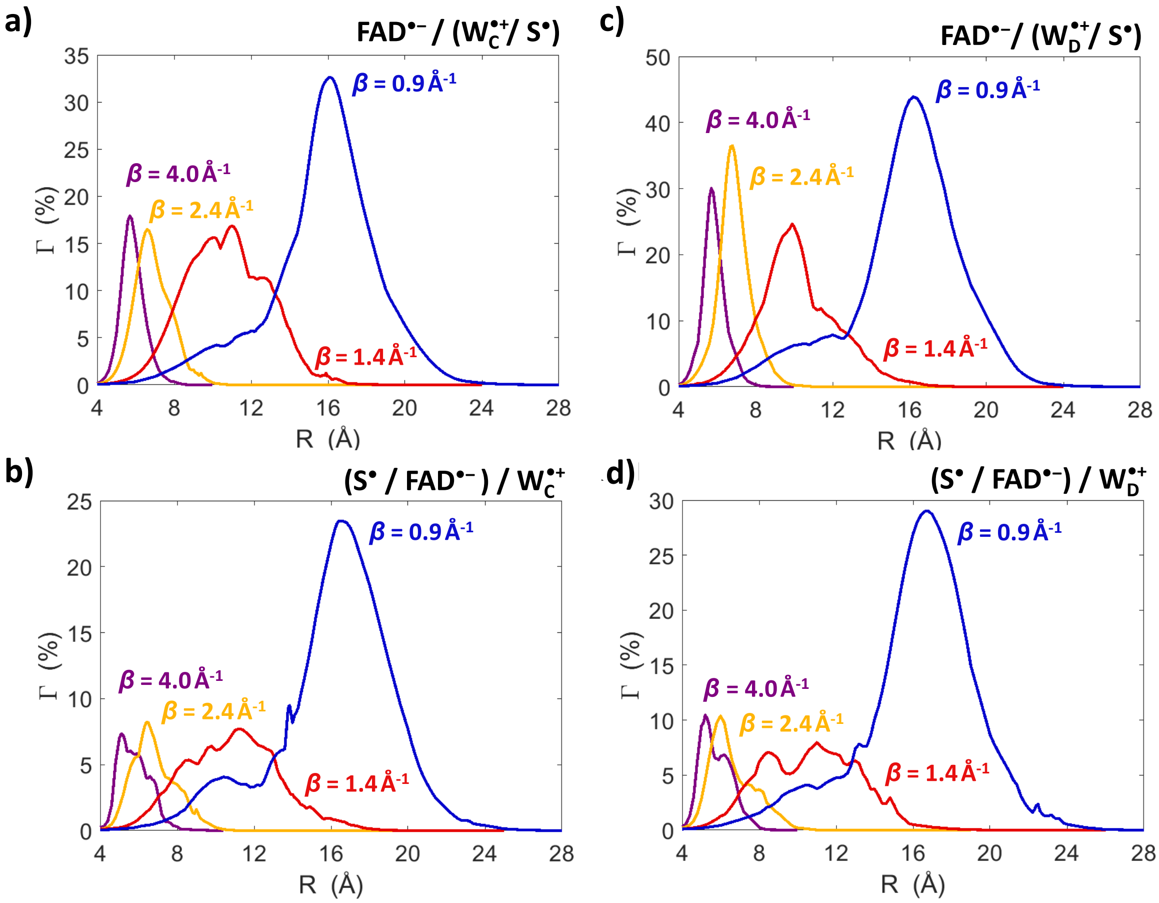

Figure S8: Maximum relative MFE (Γ) by distance from the scavenged radical $S^{\bullet}$, for models of $FAD^{\bullet-}/W^{\bullet+}$ primary-pair types in *Dm*Cry based on activationless ET through four tunneling media: covalently-bound (blue), typical protein (red), "soft" vacuum (yellow), and "hard" vacuum (purple). Subfigures **a** and **c** show MFEs from simulations wherein $W^{\bullet+}$ was scavenged by $S^{\bullet}$, whereas subfigures **b** and **d** show results for $FAD^{\bullet-}$ scavenged by $S^{\bullet}$. Tunneling decay parameters are indicated by colour, in figure. Brackets are used in the subfigure labels to indicate the radicals involved in the scavenging process. Note that the anisotropic MFE becomes negligible in the large scavenging-radius limit as ET with the scavenger approaches zero and the primary radical recombination has been neglected.

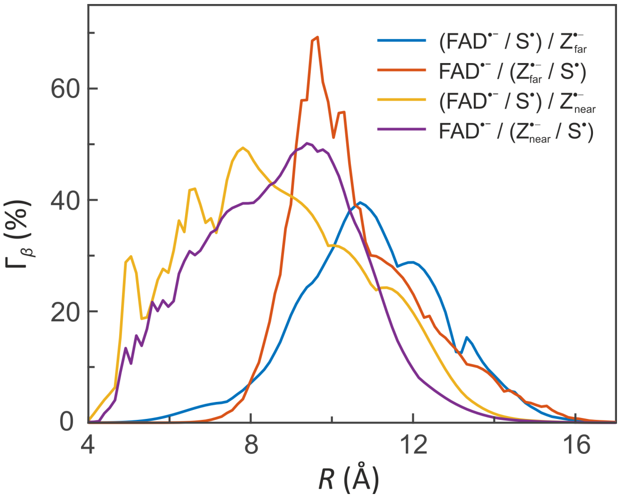

Figure S9: Maximum relative MFE (Γ) by distance from the scavenger radical $S^\bullet$, for models of $FAD^{\bullet-}/Z^{\bullet-}$ born with random spin configuration (*i.e.*, as F-pairs), assuming activationless ET through protein media ($\beta = 1.4\,\text{Å}$). The legend specifies the identity of the scavenged radical, where brackets indicate the pair involved in the scavenging process. Note that the anisotropic MFE becomes negligible in the large scavenging-distance limit as the ET with the scavenger approaches zero and the primary radical recombination has been neglected.

Scavenging-mediated Absolute Anisotropies in *Cl*Cry4

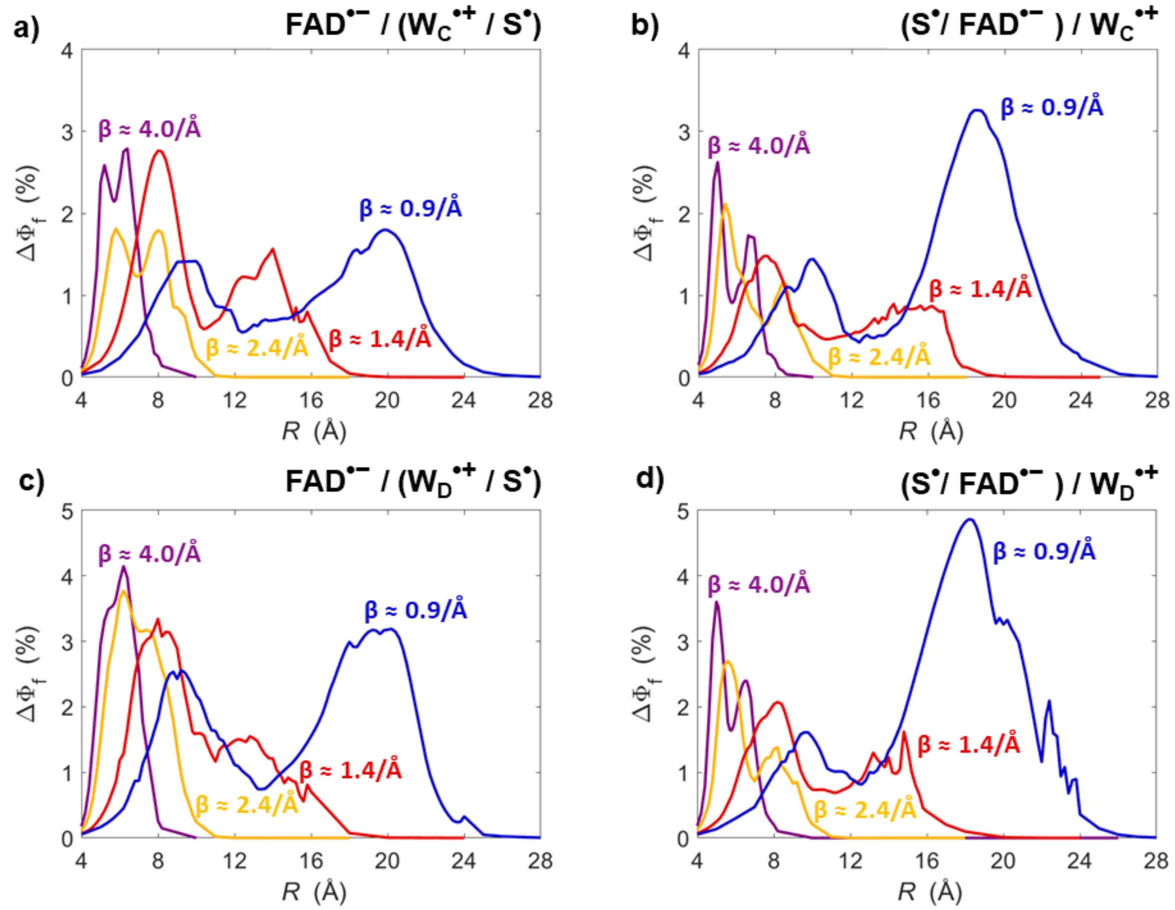

Figure S10: Maximum absolute MFE ($\Delta\Phi_\mathrm{f}$) by scavenger distance R from the scavenged radical $S^\bullet$, for models of $FAD^{\bullet-}/W^{\bullet+}$ primary-pair types in *Cl*Cry4 based on activationless ET through four tunneling media: covalently-bound (blue), typical protein (red), "soft" vacuum (yellow), and "hard" vacuum (purple). Subfigures **a** and **c** show MFEs from simulations wherein $W^{\bullet+}$ was scavenged by $S^\bullet$, whereas subfigures **b** and **d** show results for $FAD^{\bullet-}$ scavenged by $S^\bullet$. Tunneling decay parameters are indicated by colour, in figure. Brackets in the subfigure labels indicate which radical is involved in the scavenging process. Note that the anisotropic MFE becomes negligible in the large scavenging-radius limit as ET with the scavenger approaches zero, and that the primary radical recombination has been neglected.

Free Scavenger-mediated Absolute Anisotropies

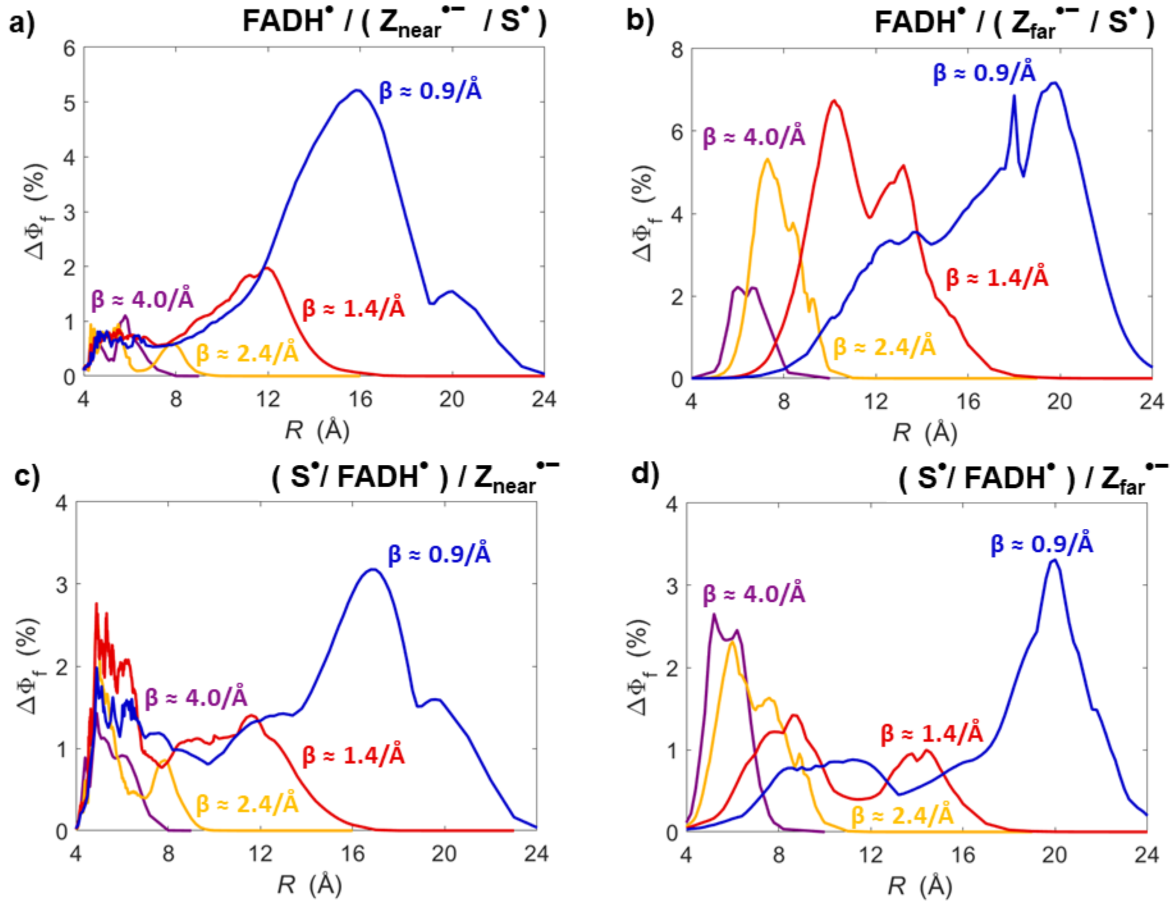

Figure S11: Maximum absolute MFE ($\Delta\Phi_f$) by distance R from the scavenger $S^\bullet$ for each $FADH^\bullet/Z^{\bullet-}$ model, based on activationless ET through four tunneling media: covalently-coupled (blue), typical protein (red), "soft" vacuum (yellow), and "hard" vacuum (purple). Subfigures **a** and **b** show results from simulations of $Z^{\bullet-}$ scavenged by $S^\bullet$, whereas subfigures **c** and **d** show MFEs from simulations of $FADH^\bullet$ scavenged by $S^\bullet$. Tunneling decay parameters are indicated by colour, in figure. Brackets are used in the subfigure labels to indicate which radical is being scavenged. These free radical predictions may be similar to those for *Cl*Cry4.

Scavenging-mediated Absolute Anisotropies in *Dm*Cry

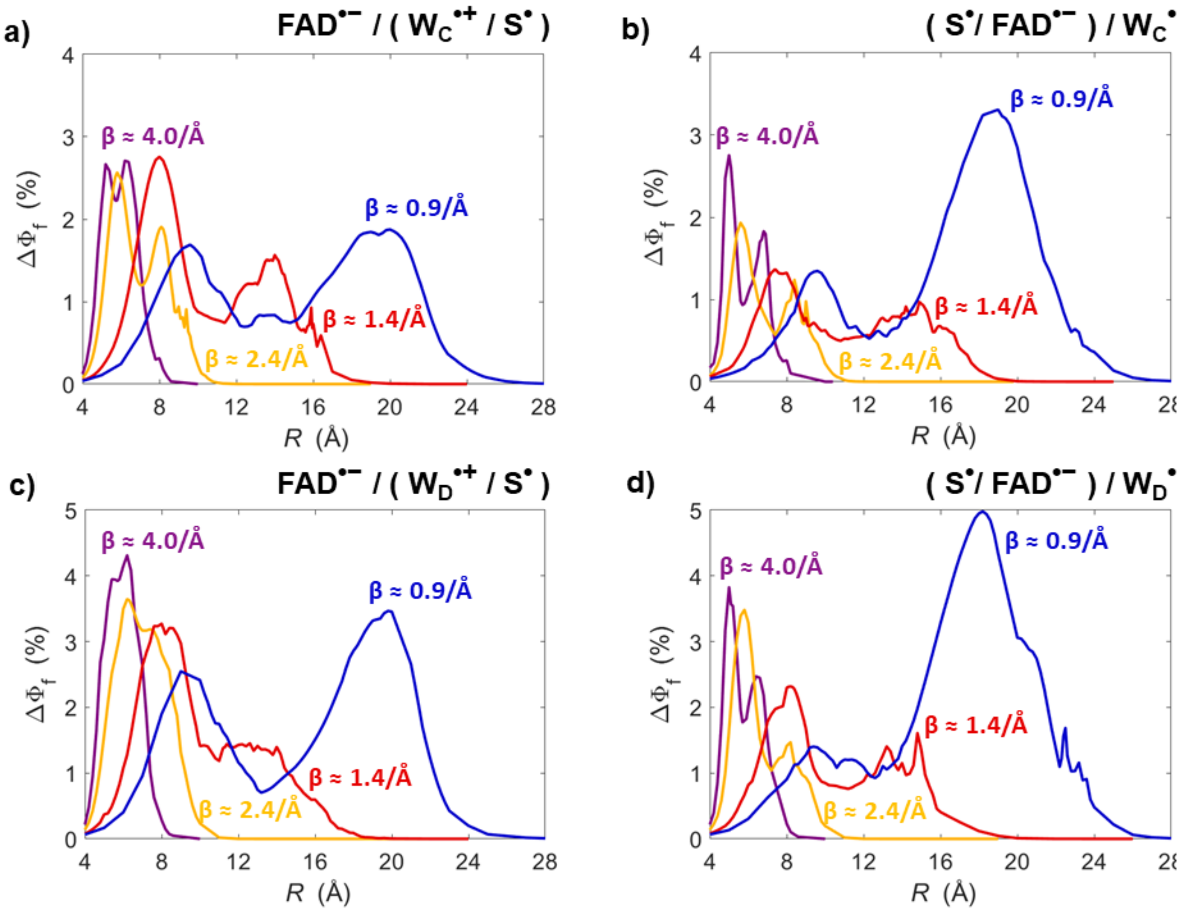

Figure S12: Maximum absolute MFE ($\Delta\Phi_f$) by scavenger distance R from the scavenged radical $S^\bullet$, for models of $FAD^{\bullet-}/W^{\bullet+}$ primary-pair types in *Dm*Cry, based on activationless ET through four tunneling media: covalently-bound (blue), typical protein (red), "soft" vacuum (yellow), and "hard" vacuum (purple). Subfigures **a** and **c** show MFEs from simulations wherein $W^{\bullet+}$ was scavenged by $S^\bullet$, whereas subfigures **b** and **d** show results for $FAD^{\bullet-}$ scavenged by $S^\bullet$. Tunneling decay parameters are indicated by colour, in figure. Brackets in the subfigure labels indicate which radical is involved in the scavenging process. Note that the anisotropic MFE becomes negligible in the large scavenging-radius limit as ET with the scavenger approaches zero, and that the primary radical recombination has been neglected.

Description of scavenger locations leading to enhanced Relative MFEs for *Dm*Cry

Scavenging of $\mathrm{TrpH}^{\bullet+}$ by Radical $\mathrm{S}^{\bullet}$

For the $\mathrm{FAD}^{\bullet-}$ / ($\mathrm{W}_{\mathrm{C}}^{\bullet+}$ / $\mathrm{S}^{\bullet}$) reaction in *Dm*Cry the through-vacuum models predicted optimal scavenger locations placed within 7 Å of W_{C}, just outside the protein surface, with a maximum MFEs of 18% (for $\beta = 4.0\,\text{Å}^{-1}$) in close proximity to Ser396, and bimodal maxima of 16% (for $\beta = 2.8\,\text{Å}^{-1}$) for scavengers located 5 Å apart, at positions either abutted next to Met331 or tucked between Pro341 and Lys344. Likewise, the typical protein-coupled model predicted bimodal maxima approaching 17% for scavengers placed in positions 14 Å apart and just outside the protein surface, either 10 Å from W_{C} (near Lys344), or 11 Å away from W_{C} (near Leu349). The covalently-bridged tunneling model predicted MFEs $> 30\%$ for more-distant scavengers located about 7 Å from the protein surface, approximately equidistant from the protruding residues Glu332 and Lys344.

Simulations of the $\mathrm{FAD}^{\bullet-}$ / ($\mathrm{W}_{\mathrm{D}}^{\bullet+}$ / $\mathrm{S}^{\bullet}$) models in *Dm*Cry predicted maxima in the MFEs $> 20\%$ for all four tunneling parameters considered. The two "vacuum-like" tunneling models predicted sharp maxima with MFEs of 30% ($\beta = 4.0\,\text{Å}^{-1}$) and 36% ($\beta = 2.8\,\text{Å}^{-1}$), both within 7 Å of W_{D} and 1.2 Å apart from one another, co-located in between residues Met506 and Met509 just inside the protein surface. Through-protein simulations predicted maxima just outside the protein surface, with maximum MFEs of 24% (for $\beta = 1.4\,\text{Å}^{-1}$) adjacent to Met506, and 44% (for $\beta = 0.9\,\text{Å}^{-1}$) above the protein surface between Arg513 and the segment connecting Arg430, Glu429, and Phe428 (near the protein C-terminus).

Scavenging of $\mathrm{FAD}^{\bullet-}$ by Radical $\mathrm{S}^{\bullet}$

For the ($\mathrm{S}^{\bullet}$ / $\mathrm{FAD}^{\bullet-}$) / $\mathrm{W}_{\mathrm{C}}^{\bullet+}$ system in *Dm*Cry the three weakest tunneling models consistently predicted maximum MFEs of 7% or 8%. Weakly-coupled models predicted optimal scavenger locations inside the protein fold, in direct contact with the FAD itself.

Models of scavenging *via* tunneling through typical protein were optimized for scavengers located either in contact with Leu405, or in direct proximity to an FAD phosphate (between Gln311 and His378). The covalently-bridged models predicted maximum MFEs of 23% for scavengers positioned across a broad arc of locations extending 18 Å from Ile308 to Phe272.

For the ($S^\bullet$ / $FAD^{\bullet-}$) / $W_D^{\bullet+}$ reactions in *Dm*Cry the vacuum-like models predicted maximum MFEs of 10% at optimal locations within 6 Å of the FAD flavin. The typical protein model predicted a bimodal optima of 8% for scavengers 11 Å away from the FAD flavin, but 15 Å apart from each other, in contact with either Leu405 or Thr389. In comparison, covalently-bridged models predicted maximum MFEs of 23% for many possible scavenger positions along a broad arc of possible locations extending 26 Å from His13 to His307.

The FAD-scavenged model of *Cl*Cry4 also produced distinctive maximum MFEs, where a [$FAD^{\bullet-}$ / $W^{\bullet+}$] primary radical pair was assumed. The ($S^\bullet$ / $FAD^{\bullet-}$) / $W_D^{\bullet+}$ model, in particular, predicted $\Gamma > 9\%$ for a scavenger located in contact with W_A for $\beta = 1.4\,\text{Å}^{-1}$ (roughly equidistant between FAD and W_D), or likewise for a scavenger nestled between FAD and Asn391 (or $Z_{\text{near}}^{\bullet-}$) for $\beta = 4.0\,\text{Å}^{-1}$. On the other hand, the ($S^\bullet$ / $FAD^{\bullet-}$) / $W_C^{\bullet+}$ model predicted $\Gamma > 7\%$ for scavengers within close contact of Gln287 or Gln380 for $\beta = 1.4\,\text{Å}^{-1}$, or likewise for those nestled inside the FAD U-turn for $\beta = 2.8\,\text{Å}^{-1}$.

Scavenging of $Z^{\bullet-}$ by Radical $S^\bullet$

The $FADH^\bullet$ / ($Z_{\text{near}}^{\bullet-}$ / $S^\bullet$) simulations all predicted MFEs > 25% for scavengers optimally located inside the *Dm*Cry protein surface, in contact with either Asp412 ($\Gamma = 38\%$, $\beta = 4.0\,\text{Å}^{-1}$), Leu408 ($\Gamma = 29\%$, $\beta = 2.8\,\text{Å}^{-1}$), Trp 375 ($\Gamma = 29\%$, $\beta = 1.4\,\text{Å}^{-1}$), or Glu373 ($\Gamma = 36\%$, $\beta = 0.9\,\text{Å}^{-1}$). For the two weakly-coupled tunneling models, optimal scavenger locations were both within 6 Å of $Z_{\text{near}}^{\bullet-}$, whereas the protein-coupled models predicted more distant scavenger locations positioned 10 Å and 15 Å away from $Z_{\text{near}}^{\bullet-}$, respectively.

The maximum MFEs predicted using the $FADH^\bullet$ / ($Z_{\text{far}}^{\bullet-}$ / $S^{\bullet-}$) reactions showed a monotonic dependence on the type of tunneling assumed, predicting smaller MFEs for

weak couplings, and larger MFEs for stronger couplings. The hard vacuum-mediated model predicted optimal MFEs of just 5% for scavengers located 6 Å from the Z_{far} radical, inside the protein cavity (but outside the protein surface) close to Trp536 in *Dm*Cry. The "soft" vacuum-mediated model predicted optimal MFEs of 14% for locations 7 Å away from the Z_{far} radical, in the protein cavity (but outside the protein surface) in close proximity to residues Arg237 and Met266. The model of typical through-protein tunneling predicted optimal MFEs approaching 37% for locations within 10 Å of Z_{far} on the edge of the protein surface in close contact with one FAD phosphate. The covalently-bridged model predicted optimal MFEs nearing 53% for scavengers placed 15 Å away from Z_{far}, just inside the *Dm*Cry protein surface, in close proximity to residues Gln369 and Glu373.

Scavenging of FADH$^\bullet$ by Radical S$^\bullet$

Next, we consider ($S^{\bullet-}$ / FADH$^\bullet$) / $Z_{near}^{\bullet-}$ reactions schemes, where the weakly-coupled models predicted immense MFEs exceeding 65% for optimally-placed scavengers at two locations within 6 Å of the FAD flavin, near both Asn419 and Arg381 in *Dm*Cry. The protein-coupled models predicted optimal MFEs of about 25% for scavengers located in the vicinity of either Phe318 (for β = 1.4 Å^{-1}) or Gln389 (for β = 0.9 Å^{-1}). Finally, we consider the ($S^{\bullet-}$ / FADH$^\bullet$) / $Z_{far}^{\bullet-}$ reaction schemes, for which vacuum tunneling-mediated ET models predict optimal MFEs near 30% for scavengers in close proximity to the FAD flavin. The model of electron tunneling through typical protein predicted MFEs approaching 23% for scavengers placed in coordination with Tyr317, Phe318, and Trp314 in *Dm*Cry. The covalently-bridged model of ET again predicted MFEs approaching 30% for scavengers placed next to Thr386.

Summary: Of particular interest were predictions of MFEs of about 20 % for a scavenger at the protein surface (in the vicinity of Met506) reacting with W_D to form a stable (closed-shell) product. Curiously, through-vacuum tunneling models predicted that a scavenger in the vicinity of Met343 and Trp394 (W_D) could act as a scavenger of W_C producing MFEs in the range of 8 % to 10 % (assuming activationless ET *via* "soft" vacuum β = 2.8 Å^{-1}).

RF magnetic field effects

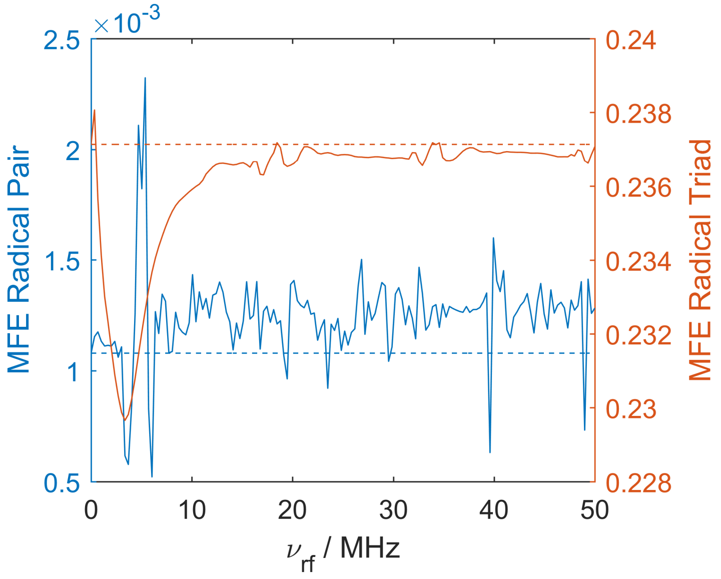

Figure S13: Sensitivity of the magnetic field effect on the reaction yield of the signalling state for the [$FAD^{\bullet-}$ / $W_D^{\bullet+}$] radical pair in *Cl*Cry4 (blue, left axis) and its cation-scavenged successor in the form of [$FAD^{\bullet-}$ / ($W_D^{\bullet+}$ / $S^{\bullet}$)] (red, right axis). For both systems, $k_f^{-1} = 3\,\mu$s. For the RPM-model $k_b^{-1} = 3\,\mu$s while the corresponding rate was neglected in the triad model, as we have discussed in the main manuscript. The scavenger radical $S^{\bullet}$ has been assumed at the location of maximal sensitivity at a distance $R = 9.9\,\text{Å}$ from the $W_D^{\bullet+}$ for which $\Gamma = 24.1\,\%$ is realized in the absence of RF fields. The RPM model only provides $\Gamma = 0.13\,\%$ The RF perturbation was assumed to have amplitude $5\,\mu$T and be oriented parallel to the molecular x-axis. The MFE is here assessed in terms of $2(Y_{\text{max}} - Y_{\text{min}})/(Y_{\text{max}} + Y_{\text{min}})$, where $Y_{\text{max,min}}$ is the yield evaluated for the static magnetic field direction of maximal/minimal reaction yield in the absence of the RF perturbation, *i.e.* in terms of the relative change of the yield at these two selected orientations of the geomagnetic field. To elicit a RF magnetic field sensitivity like that observed in behaviour experiments, a significantly longer lifetime or amplified RF field amplitude needed to be assumed for both the RPM-model and the model suggested here. Horizontal dashed lines indicate the MFE at zero RF frequency.

References

(1) Babcock, N. S.; Kattnig, D. R. Electron–electron dipolar interaction poses a challenge to the radical pair mechanism of magnetoreception. *J. Phys. Chem. Lett.* **2020**, *11*, 2414–2421.

(2) Levy, C.; Zoltowski, B. D.; Jones, A. R.; Vaidya, A. T.; Top, D.; Widom, J.; Young, M. W.; Scrutton, N. S.; Crane, B. R.; Leys, D. Updated structure of Drosophila cryptochrome. *Nature* **2013**, *495*, E3–E4.

(3) Berman, H.; Henrick, K.; Nakamura, H. Announcing the worldwide protein data bank. *Nature Struct. Mol. Biol.* **2003**, *10*, 980–980.

(4) Kattnig, D. R.; Nielsen, C.; Solov'yov, I. A. Molecular dynamics simulations disclose early stages of the photo-activation of cryptochrome 4. *New J. Phys.* **2018**, *20*, 083018.

(5) Xu, J.; Jarocha, L. E.; Zollitsch, T.; Konowalczyk, M.; Henbest, K. B.; Richert, S.; Golesworthy, M. J.; Schmidt, J.; Déjean, V.; Sowood, D. J. C. et al. Magnetic sensitivity of cryptochrome 4 from a migratory songbird. *Nature* **2021**, *594*, 535–540.

(6) Atkins, C.; Bajpai, K.; Rumball, J.; Kattnig, D. R. On the optimal relative orientation of radicals in the cryptochrome magnetic compass. *J. Chem. Phys.* **2019**, *151*, 065103.
